# Identification of Novel Alleles and Structural Haplotypes of Major Histocompatibility Complex Class I and DRB Genes in Domestic Cat (*Felis catus*) by a Newly Developed NGS-Based Genotyping Method

**DOI:** 10.3389/fgene.2020.00750

**Published:** 2020-07-15

**Authors:** Masaharu Okano, Jiro Miyamae, Shingo Suzuki, Kohei Nishiya, Fumihiko Katakura, Jerzy K. Kulski, Tadaaki Moritomo, Takashi Shiina

**Affiliations:** ^1^Department of Veterinary Medicine, College of Bioresource Sciences, Nihon University, Fujisawa, Japan; ^2^Faculty of Veterinary Medicine, Okayama University of Science, Imabari, Japan; ^3^Division of Basic Medical Science and Molecular Medicine, Department of Molecular Life Science, Tokai University, Isehara, Japan; ^4^Faculty of Health and Medical Sciences, UWA Medical School, The University of Western Australia, Perth, WA, Australia

**Keywords:** domestic cat, major histocompatibility complex (MHC), polymorphism, haplotype, next generation sequencing (NGS)

## Abstract

The major histocompatibility complex (MHC) is a highly polymorphic and duplicated genomic region that encodes transplantation and immune regulatory molecules. Although it is well-known that particular MHC allelic polymorphisms and haplotypes are genetically relate to immune-mediated diseases detailed information of the cat MHC (Feline Leukocyte Antigen; FLA) genetic and haplotypic structure and diversity is limited in comparison to humans and many other species. In this study, to better understand the degree and types of allele and allelic haplotype diversity of FLA-class I (FLA-I) and FLA-DRB loci in domestic cats, we identified six expressible FLA-I loci in peripheral white blood cells by *in silico* estimation of the coding exons and NGS-based amplicon sequencing using five unrelated cats. We then used a newly developed NGS-based genotyping method to genotype and annotate 32 FLA-I and 16 FLA-DRB sequences in two families of 20 domestic cats. A total of 14 FLA-I and seven FLA-DRB were identified as novel polymorphic sequences. Phylogenetic analyses grouped the sequences into six FLA-I (FLA-E/H/K, FLA-A, FLA-J, FLA-L, FLA-O and a tentatively named FLA-E/H/K_Rec) and four FLA-DRB (FLA-DRB1, FLA-DRB3, FLA-DRB4, and FLA-DRB5) lineages. Pedigree analysis of two cat families revealed eight distinct FLA structural haplotypes (Class I – DRB) with five to eight FLA-I and two to three FLA-DRB transcribed loci per haplotype. It is evident that the eight FLA haplotypes were generated by gene duplications and deletions, and rearrangements by genetic recombination with the accumulation and/or inheritance of novel polymorphisms. These findings are useful for further genetic diversity analysis and disease association studies among cat breeds and in veterinary medicine.

## Introduction

The domestic variant of the cat species (*Felis catus*) of the family *Felidae* is thought to have originated approximately 10,000 years ago from the Libyan wild cat (*Felis lybica lybica*) of the wild cat family (Vigne et al., 2004). The domestic cat is one of the most popular companion animals of humans world-wide with a global population estimated at 600 million (Migiro, 2018). The remarkable development of breeding and veterinary technology has prolonged the life span of cats, and they are an excellent model for various human chronic and infectious diseases including human-like tumors and autoimmune diseases such as lymphoma and inflammatory bowel disease (Willard, 1999; Paulin et al., 2018). Cats also have long been used as model animals in the research fields of the cerebral nervous system and the renal urinary system (Kuwabara et al., 2010) and for viral diseases such as feline leukemia virus that is similarly to human leukemia viruses (HTLV-I and HTLV-II) (Hardy, 1984), feline immunodeficiency virus that has similar symptoms to human AIDS (Brown et al., 1994; Carpenter et al., 1996; Troyer et al., 2004, 2005), and feline infectious peritonitis virus that belongs to the same virus genus as human Coronavirus (Pearks Wilkerson et al., 2004). These and other viral diseases in cats are investigated to better understand the host-defense mechanisms against viral infections (Olmsted et al., 1992; O’Brien et al., 2006). Many of these infectious and chronic diseases and phenotypes are influenced by polymorphisms within the major histocompatibility complex (MHC) genes that are involved in transplantation (Shiina et al., 2004, 2009). However, the genetic association of such diseases with particular feline MHC (Feline Leukocyte Antigen; FLA) polymorphisms is unknown largely because polymorphism information of FLA genes is still lacking. One of the main reasons for this limitation may be the absence of appropriate technology and reagents for the comprehensive genotyping of FLA class I (FLA-I) and FLA class II (FLA-II) genes.

**FIGURE 1:**
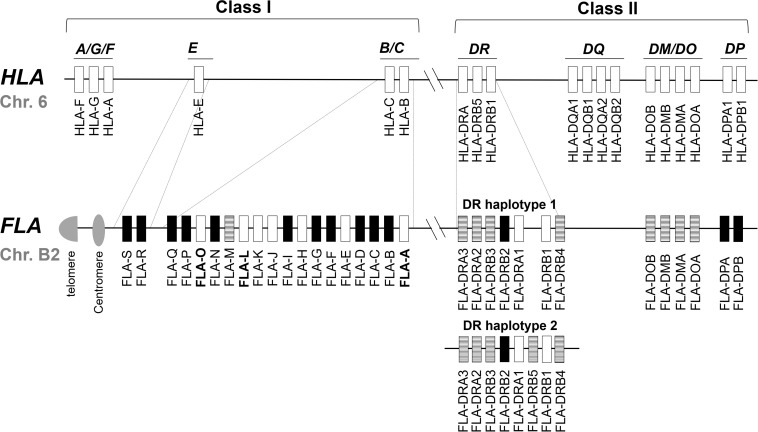
Comparative genome map of FLA and HLA genomic regions. This map shows the approximate locations of MHC genes based on the HLA (NC_000006.12) and FLA genomic information (EU153401 and Beck et al., 2001). The genomic structure of the DR haplotypes 1 is based on the genomic sequence EU153401, whereas the DR haplotype 2 is based on the description of a published report (Yuhki et al., 2008) because its nucleotide sequence has not been published as yet. White, striped and black boxes indicate transcribed genes, gene candidates and pseudogenes, respectively, after our classification in this study. In the HLA map, only the expressed genes are described. Dotted lines indicate orthologous genomic regions (E, B/C, and DR subregions) where the number of MHC genes differs between HLA and FLA. Bold letter of the gene name indicates a gene whose transcription was newly confirmed in this study.

The FLA genomic region is located on cat chromosome B2 and it is classified from the direction of the centromere to the telomere into class I, class III, and class II regions, respectively (Yuhki and O’Brien, 1988). Sequencing of the FLA genomic region has revealed 19 FLA-I and 13 FLA-II loci within the 2.98 Mb genomic sequence of accession number EU153401 (Yuhki et al., 2008) (Figure 1). In comparison with the human MHC (human leukocyte antigen; HLA) genomic region, the FLA genomic region has the following features: (1) the 19 FLA-I loci are located in genomic regions corresponding to the HLA-B, HLA-C and HLA-E subregions, (2) three FLA-DRA and five FLA-DRB loci are located in the MHC-DR subregion, but the FLA-DRB5 locus, that is not found in DR haplotype 1, is located in the genomic sequence of another MHC-DR haplotype (DR haplotype 2 in Figure 1), (3) a similar gene organization was observed in the DM/DO subregion between both species, (4) no FLA loci are found in orthologous regions corresponding to the HLA-A, HLA-A/G/F, and HLA-DQ subregions, and (5) FLA-DPA1 and FLA-DPB1 loci are located in the orthologous HLA-DP subregion, but both of them are distinct pseudogenes (Yuhki and O’Brien, 1997; Beck et al., 2001; Yuhki et al., 2008; Holmes et al., 2013) (Figure 1).

Of the 19 classified FLA-I loci, three (FLA-E, FLA-H, and FLA-K) are classical, nine (FLA-A, FLA-C, FLA-F, FLA-J, FLA-L, FLA-M, FLA-O, FLA-Q, and FLA-S) are non-classical and the other seven loci are pseudogene fragments (Yuhki et al., 2008). The FLA-E, FLA-H, and FLA-K loci were characterized as classical class I genes (class Ia) by variability index plot analysis and detailed gene expression analyses (Holmes et al., 2013). However, for the other FLA loci, the gene expression and structural characteristics such as conservation of coding sequence (CDS) is unknown, although a mRNA sequence was identified that is highly similar to FLA-J (97.2% identity with EU915358). Of the eight DR loci, two (FLA-DRA1 and FLA-DRB1) are transcribed genes (Yuhki and O’Brien, 1997) and the other five (FLA-DRA2, FLA-DRA3, FLA-DRB3, FLA-DRB4, and FLA-DRB5), although they have expressible coding exons and with mRNA sequences that are highly similar, are considered to be candidate genes because they are not identical to FLA-DRA3 (99.7% with EU915361), FLA-DRB3 (99.6% with EU916195) and FLA-DRB5 (99.6% with EU916193). The FLA-DRB2 sequence is a distinct pseudogene with only partial exons and no evidence of gene expression.

Some polymorphism analyses were performed in the FLA-I and FLA-DR loci by a PCR – sequence based typing (PCR-SBT) method using locus specific primer pairs in a sample of 12 cats that resulted with 12 allelic sequences for FLA-E, 11 for FLA-H and 10 for FLA-K (Holmes et al., 2013). On the other hand, 82 DRB allele sequences in a total of 176 cats were identified using the methods of PCR-SBT (Yuhki and O’Brien, 1997; Kennedy et al., 2002), PCR – restriction fragment length polymorphism (PCR-RFLP) (Kuwahara et al., 2000; Kuwahara et al., 2001) and PCR - reference strand-mediated conformation analysis (PCR-RSCA) (Kennedy et al., 2003). Since polymorphism analysis of the FLA genes is largely limited, the development of a new FLA genotyping method that comprehensively detects FLA polymorphisms in the FLA-I and FLA-DRB loci is required for further genetic diversity analysis among cat breeds and disease association studies in the veterinary medicine field. In this regard, a complementary DNA (cDNA)-based amplicon sequencing method by next generation sequencing (NGS) is potentially an optimal and effective procedure for high-throughput genotyping of MHC genes, FLA polymorphism analysis and detection of low-level-transcribed MHC alleles (Wiseman et al., 2009). This NGS genotyping method has been used successfully to discover and catalog a large number of MHC alleles in macaque species and other vertebrate species (Shiina et al., 2015, 2016).

In this study, to better understand the degree and types of allele and allelic haplotype diversity of FLA-I and FLA-DRB loci in domestic cats, we first elucidated the expressible FLA-I loci by *in silico* prediction of FLA-I genes and then used this information to develop the amplicon sequencing method by using primer pairs that could amplify all expressible FLA-I genes. This multi-locus analytical method was developed and used because the gene transcription of FLA-I loci except for the well characterized FLA-E, FLA-H and FLA-K loci was largely unknown. Consequently, we identified 32 FLA-I and 16 FLA-DRB allele sequences within two families of 20 domestic cats using cDNA reverse transcribed from the RNA isolated from peripheral white blood cells (WBCs). The genotyping data of FLA-E, FLA-H, and FLA-K (FLA-E/H/K) genes also was confirmed by using different primer pairs designed by Holmes et al. (2013). Phylogenetic and structural analyses of the FLA-I and FLA-DRB sequences and pedigree studies of three generations of two feline families revealed that eight FLA Class I/DRB Class II haplotypes were generated by gene duplication, accumulation of polymorphism and rearrangements by genetic recombination.

## Materials and Methods

### Animals

Peripheral white blood cells (WBCs) were obtained from 20 related domestic cats from two mongrel families (Kitayama Labes, Co. Ltd. Nagano, Japan) and five unrelated cats (four Abyssinian and one mongrel) [Animal Medical Center (ANMEC) at Nihon University]. Each family (Family 1 and Family 2) was composed of 10 cats representing three generations that were considered suitable for investigating the FLA allele and haplotype segregation. Supplementary Figure S2B provides a list of the names and gender of the twenty cats in each generation of the two families. We first used the five unrelated cats to elucidate the alleles and specificity of transcribed FLA-I loci in WBCs and then used the 20 related cats for the NGS-based genotyping of expressible FLA-I and FLA-DRB loci. The blood collection and the following study were conducted in accordance with the ethical guidelines for animal experiments specific to each location (Kitayama Labes Co., Ltd. Animal Welfare Committee and Nihon University Animal Medical Center Management and Ethics Committee).

### Research Plan

The workflow for the polymorphism analysis of FLA class I and DRB genes by NGS is shown in Supplementary Figure S1. The basic steps were: (1) *in silico* prediction of expressible FLA-I loci, (2) confirmation of FLA-I transcription and collection of the FLA-I sequences, (3) design of primer pairs for genotyping, (4) genotyping of the FLA-I and FLA-DRB genes, (5) classification of the FLA sequences, (6) evaluation of the genotyping system, (7) estimation of transcription levels, and (8) haplotyping of the FLA sequences.

### Total RNA Isolation and Reverse-Transcriptase (RT) Reaction

Total RNA was isolated from WBCs using the TRIzol reagent (Thermo Fisher Scientific, Carlsbad, CA, United States) or the NucleoSpin RNA method (Takara Bio, Shiga, Japan) according to the manufacturer’s instructions. cDNA was synthesized by oligo d(T) primer using the ReverTraAce for reverse transcriptase reaction (TOYOBO, Osaka, Japan) after treatment of the isolated RNA with DNase I (Thermo Fisher Scientific).

### PCR Primers and PCR Conditions

Five primer pairs were designed for RNA transcription analysis of FLA-I genes (1), for sub-cloning of FLA-E/H/K genes (2), for genotyping of FLA-I and FLA-DRB genes (3) and for confirmation of genotyping data in FLA-E/H/K genes (4). The primer names, the 5′–3′ nucleotide sequences, primer positions and the amplicon sizes were presented in Table 2. The exact primer locations and comparison of the primer sequences among FLA-I loci and among FLA-DRB loci are shown in Supplementary Figure S3. PCR reactions were performed with the thermal cycler GeneAmp PCR system 9700 (Thermo Fisher Scientific).

#### PCR Condition for Transcription Analysis of FLA-I Genes

The primers for transcription analysis of FLA-I genes were designed to target and amplify exons 3–4 based on eight expressible FLA-I sequences (FLA-A, FLA-E, FLA-H, FLA-J, FLA-K, FLA-L, FLA-M, and FLA-O) in the cat genome reference sequence (EU153401) (Table 2A). The 20 μL PCR amplification-reaction-volume contained 10 ng of cDNA, 1 unit of PrimeSTAR GXL DNA polymerase (Takara Bio), 4.0 μL of 5 × PrimeSTAR GXL Buffer (5 mM Mg^2+^), 1.6 μL of 2.5 mM of each dNTP and 0.4 μM of each primer. The cycling parameters were as follows: an initial denaturation of 96°C for 2 min. followed by 35 cycles of 98°C for 10 s, 55°C for 15 s, and 68°C for 30 s and a final elongation of 72°C for 2 min.

We also designed five different types of fusion primers that contained the Ion Torrent adaptors (A adaptor and P1 adaptor), 10-bp barcodes, and the FLA-I specific primers for multiplex amplicon sequencing using the Ion Torrent system and applied them for PCR amplification.

#### PCR Condition for Sub-Cloning of FLA-E/H/K Genes

The primers for sub-cloning of FLA-E/H/K genes were designed to target and amplify exons 1–8 based on FLA-E, FLA-H, and FLA-K sequences in the cat genome reference sequence (EU153401) (Table 2B). The 20 μL PCR amplification-reaction-volume contained 10 ng of cDNA, 0.4 units of KOD FX polymerase (TOYOBO), 1 × PCR buffer, 2 mM of each dNTP and 0.4 μM of each primer. The cycling parameters were as follows: an initial denaturation of 94°C for 2 min followed by 35 cycles of 98°C for 10 s, 58°C for 30 s, and 68°C for 1 min and a final elongation of 72°C for 2 min.

#### PCR Condition for Genotyping of FLA-I and FLA-DRB Genes

The primers for genotyping FLA-I genes were designed to target and amplify exons 2–3 based on known and newly determined FLA-I sequences from the seven transcribed FLA-I loci (FLA-A, FLA-E, FLA-H, FLA-J, FLA-K, FLA-L, and FLA-O) that were confirmed by transcribed analysis in WBCs (Table 2C). The 20 μL amplification-reaction-volume contained 10 ng of cDNA, 0.4 units of KOD FX polymerase (TOYOBO), 1 × PCR buffer, 2 mM of each dNTP and 0.4 μM of each primer. The cycling conditions were as follows: an initial denaturation of 96°C for 2 min. followed by 35 cycles of 98°C for 10 s, 56°C for 30 s, and 68°C for 30 s and a final elongation of 72°C for 2 min.

The primers for genotyping of FLA-DRB genes were designed to specifically target exons 1–4 based on known sequences from the four FLA-DRB loci (FLA-DRB1, FLA-DRB3, FLA-DRB4, and FLA-DRB5) (Table 2D). The 20 μL PCR amplification-reaction-volume contained 10 ng of cDNA, 1 unit of PrimeSTAR GXL DNA polymerase (Takara Bio), 4.0 μL of 5 × PrimeSTAR GXL Buffer (5 mM Mg^2+^), 1.6 μL of 2.5 mM of each dNTP and 0.4 μM of each primer. The cycling conditions were as follows: an initial denaturation of 96°C for 2 min followed by 40 cycles of 98°C for 10 s, 55°C for 30 s and 68°C for 30 s and a final elongation of 72°C for 2 min.

We also designed 20 different types of fusion primers that contained the Ion Torrent adaptor (A adaptor and P1 adaptor), a 10 bp barcodes, and FLA-I or FLA-DRB specific primers for multiplex amplicon sequencing using the Ion Torrent system and applied them for PCR amplification.

#### PCR Conditions for Confirmation of Genotyping Data in FLA-E/H/K Genes

The FLA-I_Umultiple_F/R primers designed by Holmes et al. (2013) were used to confirm the genotyping data of FLA-E/H/K genes obtained from the amplicon sequencing (Table 2E). The primers targeted exons 1–4 to amplify the FLA-E/H/K genes. The 20 μL amplification-reaction-volume contained 10 ng of cDNA, 0.4 units of KOD FX polymerase (TOYOBO), 1 × PCR buffer, 2 mM of each dNTP and 0.4 μM of each primer. The cycling conditions were as follows: an initial denaturation of 96°C for 2 min followed by 35 cycles of 98°C for 10 s, 58°C for 30 s, and 68°C for 1 min and a final elongation of 72°C for 2 min.

### NGS Using the Ion Torrent System

#### Purification and Quantification of the Amplicons

The amplicons were purified by the Agencourt AMPure XP (Beckman Coulter, Fullerton, CA, United States) and quantified by the PicoGreen assay (Thermo Fisher Scientific) with the Fluoroskan Ascent micro-plate fluorometer (Thermo Fisher Scientific). The amplicons amplified with the fusion primers were mixed at equimolar concentrations and diluted according to the manufacturer’s recommendations (Thermo Fisher Scientific).

#### Construction of NGS Library Using the Ion Torrent System

Preparation of barcoded-library DNA samples from the amplicons that were amplified by the primer pairs to confirm the genotyping data in FLA-E/H/K genes, fragmentation, DNA library amplification, measurement of DNA size and quantitation were performed according to the previous report (Ozaki et al., 2015).

#### Emulsion PCR, Sequencing Run and Data Processing

Emulsion PCR (emPCR) was performed with the Ion PGM Template IA 500 Kit (Thermo Fisher Scientific) and GeneAmp PCR system 9700 (Thermo Fisher Scientific) for transcription analysis of FLA-I genes and with the Ion 520 and 530 Kit-OT2 and OneTouch 2 instrument (Thermo Fisher Scientific) for genotyping of FLA-I and FLA-DRB genes and confirmation of genotyping data in FLA-E/H/K genes. After the emulsion PCR, the beads carrying the single-stranded DNA templates were enriched with the Ion OneTouch Enrichment System (Thermo Fisher Scientific) according to the manufacture’s recommendation. Sequencing was performed using the Ion PGM Hi-Q View Sequencing Kit and Ion 316 Chip Kit (Thermo Fisher Scientific) for transcription analysis and using the Ion S5 Sequencing Kit and Ion 520/530 Chip Kit (Thermo Fisher Scientific) for genotyping of FLA-I and FLA-DRB genes and confirmation of genotyping data in FLA-E/H/K genes.

The raw data processing and base-calling, trimming and output of quality-filter sequence reads that were binned on the basis of the Ion Xpress Barcodes into separate sequence fastq files, were all performed by the Torrent Suite 4.2.1 (Thermo Fisher Scientific) for the transcription analysis and by the Torrent Suite 5.6.0 (Thermo Fisher Scientific) for the genotyping with full processing for shotgun analysis.

### Identification and Assignment of FLA-I and FLA-DRB Allele Sequences

Known and novel FLA-I and FLA-DRB allele sequences from the output reads were identified by *de novo* assembly and mapping analyses. At first, sequence reads with average quality values (QVs) of 10 or more were extracted for *de novo* assembly analysis using the PRINSEQ software ver. 0.20.3 lite (Schmieder and Edwards, 2011). Primer sites were trimmed from the extracted reads using the Sequencher ver. 5.0.1 DNA sequence assembly software (Gene Code, Ann Arbor, MI, United States). The trimmed reads were aligned using the Sequencher 5.0.1 with minimum match percentage “99 or 100%” and minimum overlap length “20” parameters and using the AmpliSAS software (Sebastian et al., 2016) with “454/IonTorrent Technology” parameters. We searched for novel FLA nucleotide sequences and splice variants using consensus sequences that were composed of 10 or more reads per individual cat sample; and by mapping the output reads with “98–99%” matching parameter and “200–372 bp” of minimum overlap length parameter against all FLA allele sequences released in the NCBI database and all FLA allele sequence candidates constructed by the *de novo* assembly using the GS Reference Mapper Ver. 3.0 (Roche, Basel, Switzerland). Finally, the FLA alleles were assigned and annotated by remapping the output reads as “100%” matching parameter and “150 bp” of minimum overlap length parameter against all the collected FLA sequences using the GS Reference Mapper Ver. 3.0 (Roche).

### Sub-Cloning and Sanger Sequencing

PCR amplicons amplified by the primer pair for sub-cloning of FLA-E/H/K genes were cloned into the pTA2 cloning vector with the TA cloning kit according to the protocol provided by the manufacturer (TOYOBO) and sequenced by using the ABI3130 genetic analyzer (Thermo Fisher Scientific) in accordance with the protocol of the Big Dye terminator method. To avoid reporting PCR and sequencing artifacts generated by polymerase errors, 21–52 clones per cat were sequenced.

### Post Sequence Analyses

Comparative nucleotide and translated amino acid sequences were analyzed using the GENETYX software (Software Development Co. Ltd., Japan). Nucleotide similarities within the NCBI database were searched by BLAST^1^. Prediction of exon–intron structure and conservation of splice sites was performed by the GeneScan program with the default setting (Burge and Karlin, 1998). Multiple sequence alignments were created using the ClustalW Sequence Alignment program of the Molecular Evolution Genetics Analysis software7 (MEGA7) (Kumar et al., 2016). Phylogenetic trees of the FLA-I and FLA-DRB genes were constructed by the Neighbor-joining (NJ) method in MEGA7 (Saitou and Nei, 1987) using novel and known 105 amino acid (aa) sequences (FLA-I genes) and 124 aa sequences (FLA-DRB). The aa sequences in the phylogenetic analyses were translated from the following FLA-I and FLA-DRB nucleotide sequences: 32 FLA-I and 16 FLA-DRB identified in this study, seven FLA-I and three FLA-DRB sequences derived from the cat genome reference (EU153401) and canine MHC class I (DLA-88, NM_001014767) and class-II (DLA-DRB1, NM_001014768) as out-groups. The NJ trees were constructed by p-distance model and assessed using 2,000 bootstrap replicates.

### Nomenclature of Novel FLA Sequences and FLA Haplotypes

The novel FLA sequences identified by genotyping in the present study were deposited in the Genbank/EMBL/DDBJ databases (accession references are given in Supplementary Tables S2A,B) with temporary serial numbers (FLA-I_001 to FLA-I_013 and FLA-DRB_001 to FLA-DRB_007). The FLA nomenclature is based on that used previously by Holmes et al. (2013). The FLA haplotypes were labeled according to the haplotype nomenclature system that was used previously for the porcine MHC (SLA) (Smith et al., 2005). For example, when a cat has a haplotype (haplotype 1) in the FLA-I subregion (Hp-1.0) and a haplotype (haplotype 2) in the DRB subregion (Hp-0.2), the overall FLA haplotype “Hp-” is described as “Hp-1.2” simply by combining and separating haplotype 1 and haplotype 2 with a period in the form of “1.2”.

## Results

### *In silico* Prediction of Expressible FLA-I Genes

We used the GeneScan program to predict the coding exons of 12 previously sequenced FLA-I loci based on an exon–intron structure and conservation of splice sites (Burge and Karlin, 1998) and by comparative analysis with known MHC HLA, mouse MHC (H2) and FLA class I sequences (Supplementary Table S1). These sequences were classified as classical or non-classical loci or gene fragments according to the criteria of Yuhki et al. (2008) with FLA-E, FLA-H, and FLA-K classified as classical, and FLA-J, FLA-M, and FLA-O as non-classical (Table 1). Ten of the twelve classical and non-classical genes appear to have eight coding exons similar to known MHC class I genes such as HLA-A^∗^01:01:01:01 (NM_001242758) and H2-K1 (L23495) (Supplementary Table S1). In fact, the FLA-A and FLA-L loci were limited to five and seven coding exons, respectively, because of translation stop codons. The nucleotide length of exons 2 and 3, encoding the α1 and α2 domains that play an important role in antigen presentation, were conserved in these eight FLA-class I sequences, similarly to the known classical FLA class I loci (Supplementary Table S1). However, four of the non-classical loci, FLA-C, FLA-F, FLA-Q, and FLA-S, appear to be pseudogenes with only partial coding exons that do not have typical MHC domain structures (Supplementary Table S1) and therefore were discarded from further analysis. Hence, only eight FLA loci, FLA-A, FLA-E, FLA-H, FLA-J, FLA-K, FLA-L, FLA-M, and FLA-O, were subjected to the subsequent transcription analysis.

**TABLE 1:** Characteristics of gene structure and classification of gene expression in FLA-class I and FLA-class II loci.

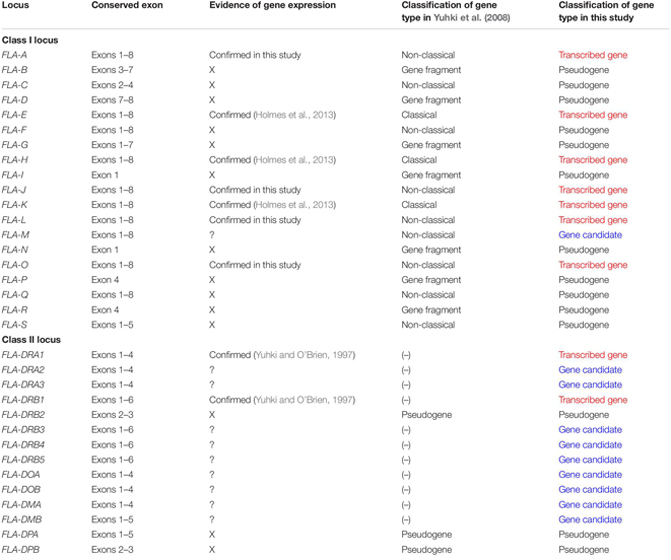

**This table shows the characteristics of gene structure based on FLA genomic sequence (EU153401) and gene type based on gene expression analyses. The “transcribed gene” was defined as a gene that has a similar exon structure and conservation of amino acid sequence with known MHC class I and class II genes such as human and mouse, and its transcription was confirmed previously or in this study. The “gene candidate” was defined as a gene that has a similar exon structure and conservation of amino acid sequence with a known transcribed FLA gene, but with no evidence of transcription. The “pseudogene” was defined as a gene that have a defect in exon structure as compared with a known transcribed FLA gene. “?” and “X” indicate that transcription is unknown and not transcribed, respectively*.*

### Identification of FLA-I Loci Transcribed in WBCs

To identify which of the eight predicted MHC-I loci were transcribed in WBCs, we performed amplicon sequencing of cDNA derived from five unrelated cats (A6, A116, A165, A176, and A214) using a primer pair designed for exons 3–4 (Table 2A). The *de novo* assembly analysis and mapping analysis were performed using the output reads, and nucleotide similarity analysis was performed between the consensus sequences (FLA-I sequences) and the cat genome reference sequence (EU153401). In total, 20 FLA-I sequences (FLAexp01 – FLAexp20) were identified in the five cats that matched with FLA-I sequences from the cat genome reference (EU153401) with a nucleotide similarity of 94–100% (Table 3). Nine to 15 FLA-I sequences (10 sequences on average) were identified per cat. Of these FLA-I sequences, three were a perfect match (100%), three were highly similar (99%), and six showed similarities of 94–98% with FLA-E/H/K-like sequences of the cat genome reference (Table 3). In addition, a perfect match or a high similarity of 99%, were obtained between seven of our sequences and the FLA-A, FLA-J, FLA-L, and FLA-O sequences of the cat genome reference, whereas one sequence was 97% similar to the FLA-J gene (Table 3). Five loci (FLA-E, -J, -K, -L, and -O) were transcribed in cat A214, six (FLA-A, -E, -J, -K, -L, and -O) in A176, and seven (FLA-A, -E, -H, -J, -K, -L, and -O) in A6, A116 and A165 (Table 3). None of the transcribed FLA-I sequences in the five cats, were highly similar to the FLA-M locus nor to the FLA-H and/or FLA-A loci in cat A176 and cat A214 (Table 3). Although the transcribed sequences FLAexp17 and FLAexp19 showed 100 and 99% nucleotide similarities, respectively, with the reference FLA-O locus (Supplementary Table S1), they had the same nucleotide length as the other FLA-I loci (252 bp, see Table 2A), and the 38-bp shorter splice variants (FLAexp18 and FLAexp20) (Table 3). These splice variants were identified at the 3′-end of exon 3 in the FLAexp18 sequence of cats A6 and A165 and the FLAexp20 sequences of all 5 cats, and they appear to contribute to a frameshift in the translated amino acid sequences in comparison to the predicted FLA-O coding exons (Supplementary Table S1). In addition, two to five FLA-E/H/K-like sequences were identified in the five cats by subcloning, and all of them were identical with the FLA-E/H/K-like sequences derived from amplicon sequencing in our investigation of the detection sensitivity of transcribed sequences between the amplicon sequencing and conventional subcloning methods. However, the FLA-A, -J, -L, -O, and -M like sequences were not detected by the subcloning method because their transcription levels were much lower than those transcribed by the FLA-E/H/K loci (Table 3). Nevertheless, taken together, at least seven FLA-I loci (FLA-A, FLA-E, FLA-H, FLA-J, FLA-K, FLA-L, and FLA-O) were transcribed in WBCs, and therefore these loci, together with the FLA-DRB genes, were targeted for the following genotyping analyses.

**TABLE 2:** Primer information used for this study.

Primer name	Primer sequence (5′–3′)	Primer position	Primer length	Product length	Analyzed length	References
**(A) For FLA-I transcribed analysis**						
FLA-I_exp_F-1	RGATTACATCGCCCTGAAC	Exon 3	19 bp	290 bp	252 bp	This study
FLA-I_exp_F-2	RGATTACATCACCCTGAAC	Exon 3	19 bp			This study
FLA-I_exp_F-3	RGATTACATCTCCCTGAAC	Exon 3	19 bp			This study
FLA-I_exp_R	GCCAGGTYRGGGTGATCTC	Exon 4	19 bp			This study
**(B) For sub-cloning of FLA-E/H/K genes**						
FLAI_CDS_F	CTCCTGAGACTCACATTTCTCC	Exon 1	22 bp	1163 bp	1122 bp	This study
FLAI_CDS_R	CAGATCCTGCATCGCTCAG	Exon 8	19 bp			This study
**(C) For genotyping of FLA-I genes**						
FLA-I_F2	GTSGGCTACGTRGACGACA	Exon 2	19 bp	353 bp	316 bp	This study
FLA-I_R2m	ATCTGCGCHGCSGTGTCC	Exon 3	18 bp			This study
**(D) For genotyping of FLA-DRB genes**						
FLA-DRB_F	YCTKGATGRCAGCTCTGATG	Exon 1	20 bp	412 bp	372 bp	This study
FLA-DRB_R	GAGCAGACCARGAGGTTGTG	Exon 4	20 bp			This study
**(E) For confirmation of genotyping data in FLA-E/H/K genes**						
FLA-I_Umultiple_F	GTGCTCCTGCTGCTGTTG	Exon 1	18 bp	827 bp	789 bp	Holmes et al., 2013
FLA-I_Umultiple_R	TGGCACGTGTATCTCTGCTC	Exon 4	20 bp			Holmes et al., 2013

**TABLE 3:** FLA-I allele sequences and their nucleotide similarities derived from the subcloning and amplicon sequencing methods using five unrelated cats.

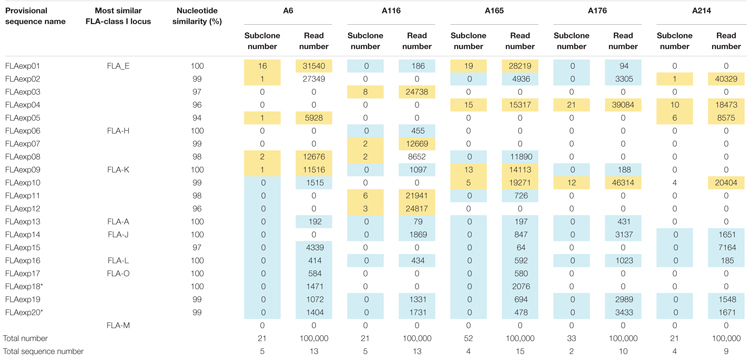

The nucleotide similarity was calculated by comparing with the FLA genomic sequence (EU153401). Yellow and blue backgrounds indicate FLA-I allele sequences detected by both the subcloning and amplicon sequencing methods and by the amplicon sequencing method only, respectively. *FLAexp18 and FLAexp20 show splice variants of FLAexp17 and FLAexp19, respectively.

### Genotyping in FLA-I and FLA-DRB Genes Using 20 Cats From Two Families

**FIGURE 2:**
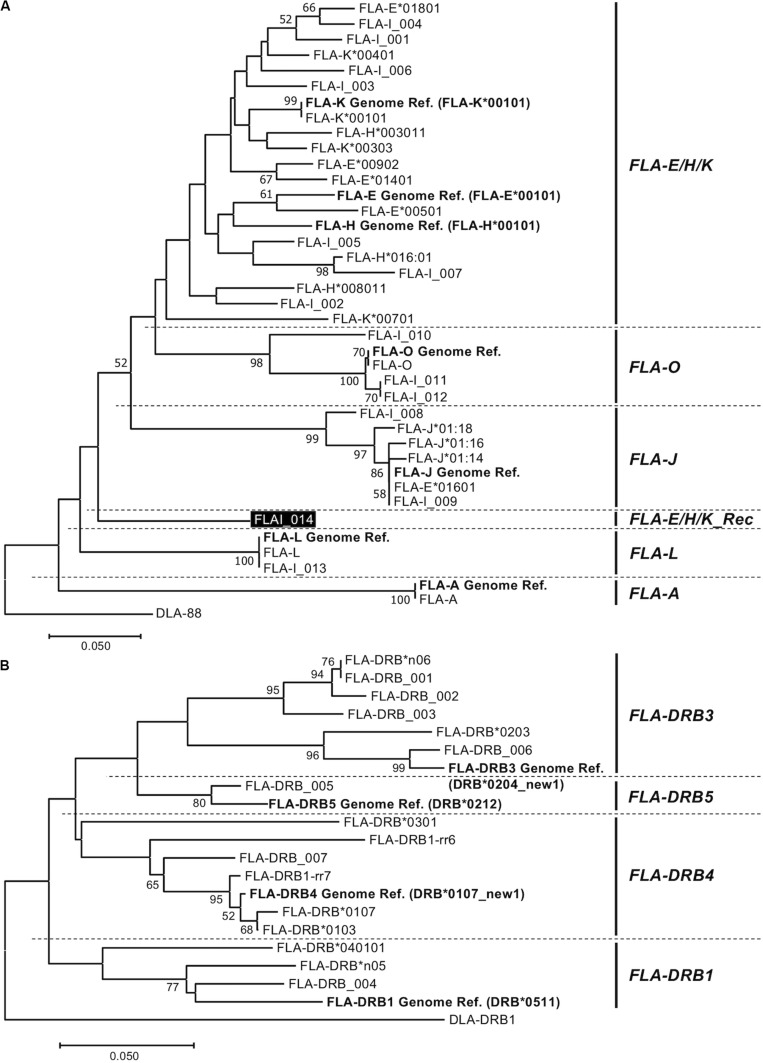
Amino acid sequence-based phylogenetic trees of FLA-I **(A)** and FLA-DRB **(B)** sequences by the Neighbor joining method. The trees were constructed by the neighbor-joining method. Numbers at branches indicate bootstrap values over 50. **(A)** FLA-I tree based on 40 amino acid sequences, 32 FLA-I translated from the nucleotide sequences generated in this study, seven FLA-I reference sequences (EU153401) (bold letters) and DLA-88 (NM_001014767) that was used as an outgroup sequence. The FLA-I_014 in FLA-E/H/K_Rec lineage is indicated by a black background and white letters. **(B)** FLA-DRB tree based on 21 amino acid sequences, 16 FLA-DRB translated from nucleotide sequences that were identified in this study, five FLA-DRB reference sequences (EU153401 and U51506) (bold letters) and a DLA-DRB1 sequence (NM_001014768) that was used as an outgroup.

The FLA-I and FLA-DRB genes transcribed in WBC samples of 20 cats from 2 families were genotyped by NGS of amplified cDNA using a primer pair designed for exons 2–3 of the FLA-I genes and for exons 1–3 of the DRB genes (Tables 2C,D). A total of 32 FLA-I and 16 FLA-DRB sequences were identified by *de novo* assembly and mapping analysis using the 316 and 372 bp regions with the primer sequence removed, respectively. Of the perfectly matched sequences, 18 were matched with known FLA-I and nine with known FLA-DRB sequences. There were also 14 novel FLA-I (tentatively named FLA-I_001 – FLA-I_014) and seven FLA-DRB (tentatively named FLA-DRB_001 – FLA-DRB_007) sequences. Of the total of 48 FLA, four combinations, FLA-I_011 and FLA-I_012, FLA-E^∗^01601 and FLA-I_009, FLA-L and FLA-I_013 and FLA-DRB^∗^n06 and FLA-DRB_001, had identical amino acid sequences accompanied by synonymous substitutions (Figure 2). On average, seven to 14 FLA-I sequences and two to six FLA-DRB sequences were identified per cat (Supplementary Tables S2A,B).

### Phylogenetic Analyses Among the FLA-I and Among FLA-DRB Sequences

To classify phylogenetic relationships among the 32 FLA-I sequences and separately among the 16 FLA-DRB sequences, phylogenetic analyses were performed using the amino acid sequences (105 aa in FLA-I and 124 aa in FLA-DRB) that were translated from the transcribed FLA sequences in this study and the FLA reference sequences (EU153401 and U51506). The phylogenetic tree divided the FLA-I and FLA-DRB sequences into five (FLA-E/H/K, FLA-A, FLA-J, FLA-L, and FLA-O) and four (FLA-DRB1, FLA-DRB3, FLA-DRB4, and FLA-DRB5) separate lineages, respectively (Figure 2). In the FLA-I tree, 31 sequences, excluding FLA-I_014, were classified further into the following 5 distinct lineages: one with FLA-A, 18 with FLA-E/H/K, six with FLA-J, two with FLA-L and four with FLA-O (Figure 2A). The 18 FLA-E/H/K-like sequences were grouped within one intermingled lineage (FLA-E/H/K lineage) due to close relationships between the sequences such as between FLA-K^∗^00401 and FLA-E Genome Ref. and between FLA-H^∗^003011 and FLA-K^∗^00303. Moreover, the tree infers that the FLA-I_014 diverged before the generation of the FLA-J lineage and after the generation of the FLA-L lineage (Figure 2A). Our BLAST search and phylogenetic analyses strongly suggest that the sequence has a chimeric structure because 124 bp of the 316 bp were perfectly matched with 12 FLA-I sequences including FLA-E^∗^00902 (KC763019), while the 191 bp were perfectly matched with three nucleotide sequences including K^∗^00801 (KC763045). We could not determine the origin of one bp that locates between the two segments (Supplementary Figure S4). Therefore, the sequence (lineage) was considered to be a recombinant of an allelic sequence belonging to the FLA-E/H/K lineage, and that we tentatively named as a new lineage, FLA-E/H/K_Rec. The tree also supported that FLA-E^∗^01601 was included in FLA-J lineage, and therefore, this sequence was assumed to be one of the six FLA-J lineage sequences (Supplementary Table S2A). The 16 FLA-DRB sequences in the FLA-DRB tree were classified into four distinct lineages with three sequences in the FLA-DRB1 lineage, six in FLA-DRB3, six in FLA-DRB4 and one in the DRB5 lineage (Figure 2B).

### Allelic Haplotype Structure of the FLA-I and FLA-DRB Subregions

**FIGURE 3:**
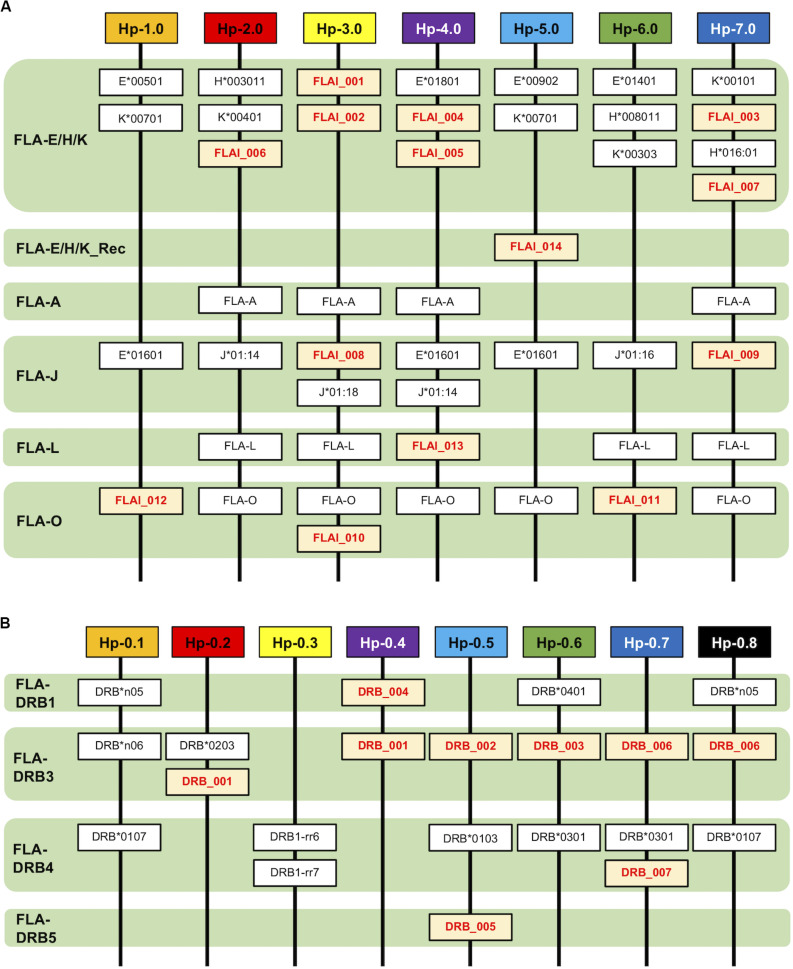
Haplotype structures of the FLA-I **(A)** and FLA-DRB **(B)** subregions. The haplotypes are composed of the different types of transcribed FLA-I and FLA-DRB loci and alleles. The lineage of the FLA-I and FLA-DRB sequences were classified and inferred from the phylogenetic analyses. Novel FLA sequences identified in this study are indicated by red letters and a yellow background.

**FIGURE 4:**
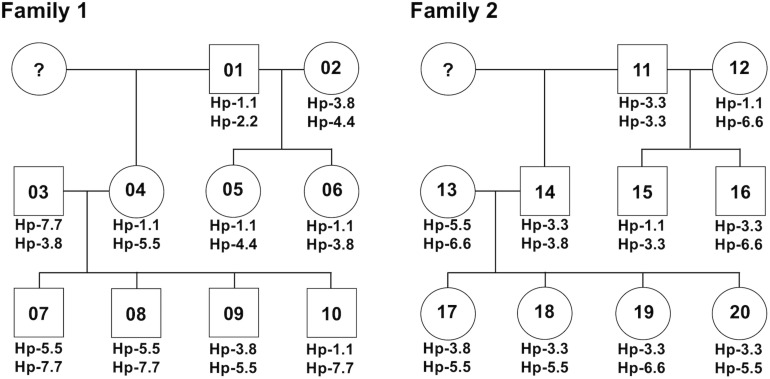
A summary for the inheritance of FLA haplotypes in two cat families. The family charts with the FLA haplotype information were summarized based on the FLA haplotype structures described in Figure 3 and Supplementary Table S3. Circles and squares indicate females and males, respectively, and numbers in the circles and squares indicate the identification number of the 20 cats. Question marks indicate individuals with unknown FLA genotypes.

After phylogenetic classification of the FLA sequences for each of FLA loci, we deduced the FLA-I and FLA-DRB haplotypes based on pedigree segregation analysis (Supplementary Figure S2). A total of seven FLA-I (Hp-1.0 – Hp.7.0) and eight FLA-DRB (Hp-0.1 – Hp.0.8) allelic haplotypes were deduced without any pedigree discrepancies (Figure 3). The FLA-I haplotypes were composed of four (Hp-1.0) to eight (Hp-3.0, Hp-4.0, and Hp-7.0) FLA-I loci. The FLA-I sequences belonging to FLA-E/H/K, FLA-J, and FLA-O phylogenetic lineages were observed in all haplotypes, but the locus copy numbers of the lineages were different for each haplotype. There were two to four loci of the FLA-E/H/K lineage, one to two loci for the FLA-J lineage and one to two loci for the FLA-O lineage (Figure 3A). In contrast, the FLA-I loci belonging to the FLA-A, FLA-L, and FLA-E/H/K_Rec lineages showed haplotype specific transcription patterns; such as Hp-2.0, Hp-3.0, Hp-4.0, and Hp-7.0 for FLA-A, Hp-2.0, Hp-3.0, Hp-4.0, Hp-6.0, and Hp-7.0 for FLA-L, and Hp-5.0 for FLA-E/H/K_Rec. On the other hand, the FLA-DRB haplotypes were composed of two to three FLA-DRB loci that showed haplotype specific transcription patterns. For example, three FLA-DRB loci from the FLA-DRB1, FLA-DRB3, and FLA-DRB4 lineages were observed in Hp-0.1, Hp-0.6, and Hp-0.8, whereas only two FLA-DRB loci from the FLA-DRB3 lineage were observed in Hp-0.2 (Figure 3B). The FLA-DRB alleles DRB^∗^0107, DRB^∗^0301, DRB^∗^n05, DRB_001, and DRB_006 were shared with different FLA-DRB haplotypes. This type of allelic sharing was also observed in K^∗^00701 of the polymorphic FLA-E/H/K lineage. Figure 4 shows the pedigree structures of FLA-I and FLA-DRB haplotype segregation in two families of twenty cats, and Supplementary Table S3 shows the haplotype structure for each cat. Eight FLA haplotypes (Hp-1.1, Hp-2.2, Hp-3.3, Hp-3.8, Hp-4.4, Hp-5.5, Hp-6.6, and Hp-7.7) were estimated from the two families used in this study, and the haplotypes were composed of seven (Hp-1.1) to 11 (Hp-3.8 and Hp-7.7) FLA loci. Of the 20 cats, male-11 with Hp-3.3 was considered to be a homozygote of the entire FLA genomic region. Of the FLA haplotypes, Hp-3.3 and Hp-3.8 share the same FLA-I haplotype, but have different FLA-DRB haplotypes, suggesting that these two haplotypes were generated by a recombination event of the genomic region between the FLA-I and FLA-DRB subregions.

### Confirmation of the FLA-I Genotyping Results

To confirm the accuracy of the genotyping results of FLA-E/H/K-like sequences derived from the NGS-based genotyping, we prepared four amplicons derived from four cats (simplified code-names: 01, 02, 07, and 13 in Figure 4) that covered all FLA-I haplotypes and one primer pair designed by Holmes et al. (2013) (Table 2E). We then performed the NGS analysis based on construction of NGS libraries using the amplicons. From a comparison of the FLA-E/H/K-like sequences obtained by this method and the previous amplicon sequencing method, the 18 FLA-E/H/K-like sequences obtained in this experiment were consistent with the sequences identified by the amplicon NGS-based genotyping method (Supplementary Table S4). Therefore, the NGS-based genotyping method that we developed for the present study was capable of genotyping the FLA-E/H/K genes with high accuracy.

## Discussion

Genome sequence analysis of the domestic cat revealed that FLA-I and DR loci are clustered within a FLA genomic region due to multiple gene duplication events. The cat MHC genomic organization and structure is similar to the dog species, and both species belong to the Carnivora order (Flynn et al., 2005; Yuhki et al., 2008). The FLA-E/H/K and FLA-DR loci have been classified as classical MHC genes, and they are thought to play an important role in disease association and genetically matching of MHC genotypes between donor and recipient in transplantation, similar to other species (Shiina et al., 2004, 2009). To further promote such research, it is necessary to develop a comprehensive genotyping method that targets those classical FLA loci. In this study, the classical FLA loci were more polymorphic than the non-classical FLA loci (Supplementary Table S5), and the nucleotide sequences between classical FLA alleles were highly similar to each other (Figure 2). Hence, it is difficult to identify and design FLA locus-specific primers for duplicated genes that have similar MHC sequence and structure (Miyamae et al., 2018). In this regard, there is only one previous report on FLA-E/H/K polymorphism analysis using FLA locus-specific primers (Holmes et al., 2013).

Recently, the NGS-based amplicon sequencing method was developed for use in a variety of species such as non-human primates (rhesus monkeys and cynomolgus monkeys), birds, reptiles, and fish to comprehensively investigate polymorphisms and transcription levels of duplicated MHC genes (Wiseman et al., 2009; Kita et al., 2012; Radwan et al., 2012; Herdegen et al., 2014; Shiina et al., 2016). Either DNA or RNA (converted to cDNA) can be used as a template for amplicon sequencing, but in this study RNA samples were used for genotyping of the FLA alleles because the advantages of using RNA samples for genotyping are that (1) transcription levels can be estimated for each of MHC alleles from the read sequence depth (Kita et al., 2012), and (2) only transcribed MHC genes are detected without contamination of amplicons originating from pseudogenes if the primer locations cross over to at least two homologous exons. Consequently, we identified FLA-I loci transcribed in WBCs by amplicon sequencing of RNA converted to cDNA after we had first used *in silico* estimations to determine the exact coding exons and then measured the transcription levels at FLA-A, FLA-F, FLA-L, and FLA-O loci (Table 3 and Supplementary Table S1). In addition, we detected 32 FLA-I and 16 FLA-DRB sequences, including 21 novel sequences, without any discrepancy in an investigation of the FLA allele and haplotype segregations in a pedigree analysis of two cat families (Figure 4 and Supplementary Table S3). Taken together, our results show that the NGS-based amplicon sequencing method using RNA samples was effective and provided accurate FLA polymorphism information (Table 3) and haplotype structures (Figure 4 and Supplementary Table S3). However, some FLA alleles might not have been amplified due to the presence of polymorphisms at the primer sites. Therefore, in the case of large-scale polymorphism analysis of various cat breeds, it will be necessary to confirm the specificity of the primers by using two kinds of primer pairs that amplify overlapping regions and account for a non-binding primer.

A possible limitation of the amplicon sequencing method using RNA samples is that it requires more reading sequences than DNA samples to genotype all MHC alleles with different levels of transcription. Thus, we developed the amplicon NGS method also to genotype the expressible FLA-I and FLA-DRB loci and demonstrated the usefulness of the NGS-based genotyping method using two families of 20 domestic cats (Supplementary Tables S2A,B). The average mapped reads of FLA-E/H/K, FLA-A, FLA-J, FLA-L, FLA-O, and FLA-E/H/K_Rec lineages were 16,444, 210, 5,069, 248, 878, and 11,820, respectively (Supplementary Table S5). The FLA-A lineage resulted in the lowest average mapped reads at approximately 1/80 of the FLA-E/H/K lineage that produced the highest average mapped reads, and, so, the transcription of the FLA-A lineage was estimated to be extremely low. Of the 20 related cats, the cat-04 (Hp-1.0/Hp-5.0) and cat-12 (Hp-1.0/Hp-6.0) did not have any reads for the FLA-A lineage. Similarly, cat-04 did not have any mapped reads of FLA-L lineage (Supplementary Table S2A). Since the mapped reads used for genotyping cat-04 and cat-05 exceed the average mapped reads (42,696 reads) by 46,589 reads and 45,490 reads, respectively, the FLA-A and FLA-L loci may be involved in transcription in a haplotype-specific manner. However, it is possible that the extremely low transcription levels transcribed by FLA-A and FLA-L loci were due to poor primer specificity rather than haplotype specificity. To solve these potential specificity problems, detailed polymorphism analysis using the NGS-based genotyping method developed in this study and gene expression analysis on the loci using a greater array of primer pairs will be necessary in future.

The average number of reads that aligned to reference nucleotides in our study was not uniform for each gene sequence and lineage (Supplementary Table S5). This was probably due to the different transcription levels transcribed by the classical and non-classical FLA genes. The FLA-E/H/K + FLA-E/H/K_Rec lineages had significantly higher average read numbers than the other lineages (16,095 read vs. 2,201 reads), and the read numbers of the FLA-DRB lineages showed a relationship of DRB5 > DRB1 > DRB3 = DRB4. In regard to the FLAI_014 allelic sequence within the FLA-E/H/K_Rec lineage, the average read numbers of eight FLAI_014 sequences (11,831 reads ± 1,410) were not significantly different (*P* = 0.06) to the read numbers (16,454 reads ± 8,136) of the 95 FLA-I sequences within the FLA-E/H/K lineage, but were significantly different (*P* = 1.2 × 10^–9^) to the read numbers (5,071 reads ± 2,649) for 56 FLA-I sequences within the FLA-J lineage (Supplementary Table S5). These read numbers suggest that the FLA-E/H/K_Rec lineage belongs to the FLA-E/H/K lineage. Although transcription levels are difficult to measure or estimate accurately from read numbers alone due to factors such as PCR efficiency or NGS differences between FLA sequences, relatively normalized read numbers based on read numbers, read length and target size appear to reflect the transcription levels of classical and non-classical FLA genes (data not shown). Further investigation is needed, but the genotyping method that we developed in this study is expected to be useful and assist to identify individual FLA genotypes and help to evaluate the different levels of gene transcription as was reported recently for HLA class I and class II gene expression (Yamamoto et al., 2020).

The domestic cat possibly emerged approximately 10,000 years ago, originating from the Libyan wild cat (Vigne et al., 2004) and then spread to Europe in the 1st century, Japan in the 8th century and North America in the 16th century (Driscoll et al., 2009). Since the 1990s, many different cat breeds became established in a short period of time (Menotti-Raymond et al., 2008). From this background history, cats have been affected by artificial positive selection during the process of pedigree breeding and may have a large number of unknown FLA alleles that show breed-specificity. Despite using only 20 related cats from two families, approximately 44% of the FLA sequences (21 sequences in total) were identified as novel in this study (Supplementary Tables S2A,B), although we did not associate these novel alleles with any particular cat breed. It is likely that our study has captured only a small fraction of the vast allelic and haplotype diversity in the domestic cat and that a large-scale collection of FLA allele sequences will be necessary to proceed smoothly in future toward disease association studies of many different cat breeds. However, on the basis of our phylogenetic analysis, we assume that FLA-I gene sequences will result in some ambiguous classifications like the FLA-I_014 rec lineage (Figure 2A). Even in this study, the FLA-E/H/K-like sequences could not be correctly classified into their particular FLA-E, FLA-H, or FLA-K loci, and, for analytical convenience, we combined the three loci into one lineage, FLA-E/H/K (Figure 2). Also, since Hp-7.0 contains four transcribed FLA-I loci belonging to the FLA-E/H/K lineage, it was difficult to classify them into one or other of the FLA-E, FLA-H, or FLA-K loci (Figure 3). Therefore, we constructed the FLA haplotype structures for future MHC classification and disease association studies based on the available information, rather than strictly classifying the FLA sequences based on each particular locus. In addition, the FLA polymorphism information provided us with a total of eight FLA haplotypes (seven FLA-I and eight FLA-DRB haplotypes). Despite the small number of haplotypes in this study, some important evolutionary events related to haplotype generation were inferred from the organization of FLA genes by copy number variation (Figure 3), recombination in exon 2 between FLA-E/H/K-like sequences (Supplementary Figure S4), recombination between FLA-I and FLA-DRB subregions (Hp-3.3 and Hp-3.8) (Figure 4), and recombination or gene conversion in the DRB subregion (Hp-0.1 and Hp-0.8) (Figure 3). It is evident from these observations that a collection of the FLA haplotype structures would lead to a better understanding of the genetic diversity system of the FLA genomic region that possibly is evolving and changing rapidly due to artificial selection (breeding) in a relatively recent evolutionary period. Therefore, when analyzing and classifying FLA haplotypes in future, it may be necessary to perform genotyping of unrelated individuals after first determining the major allele sequences and haplotype structures using families of many different breeds.

## Conclusion

We have identified and presented novel alleles for the FLA-I and FLA-DRB genes and provided some further insights into the development of the NGS-based genotyping method at the transcription level, phylogenetic relationships and haplotype structures of FLA-I and FLA-DRB alleles and haplotypes in 20 cats from two families. This FLA polymorphism and haplotype information provides a framework for future studies to interrogate the MHC in greater detail and to establish the feline MHC genetic background for the benefit of biomedical research into disease associations such as infectious disease and autoimmune diseases in veterinary medical field.

## Data Availability Statement

The novel FLA allele sequences are available in GenBank/DDBJ/ENBL-EBI DNA databases under the Accession Numbers LC534228–LC534248. We deposited our NGS data for DDBJ under accession number (DRA Accession DRA010032).

## Ethics Statement

The animal study was reviewed and approved by Nihon University Animal Medical Center Management and Ethics Committee Kitayama Labes Co., Ltd. Animal Welfare Committee.

## Author Contributions

MO, TM, and TS participated in the design of this study. MO and JM carried out most of the experiments. KN and FK interpretation of the data and proofreading the manuscript. MO, SS, JK, TM, and TS analyzed the data and wrote the manuscript. All authors checked the final version of the manuscript.

## Conflict of Interest

The authors declare that the research was conducted in the absence of any commercial or financial relationships that could be construed as a potential conflict of interest.

## Footnotes

1 http://www.ncbi.nlm.nih.gov/BLAST/
