# Supplementary material for: Identification of Novel Alleles and Structural Haplotypes of Major Histocompatibility Complex Class I and DRB Genes in Domestic Cat (Felis catus) by a Newly Developed NGS-Based Genotyping Method
Source: Front Genet. 2020 Jul 15;11:750. doi: 10.3389/fgene.2020.00750 (PMC7375346; doi:10.3389/fgene.2020.00750)
Supplement: Supplementary file 1 [file Data_Sheet_1.zip › Supplementary Figure 2.PDF]

Supplementary figure 2

(A)

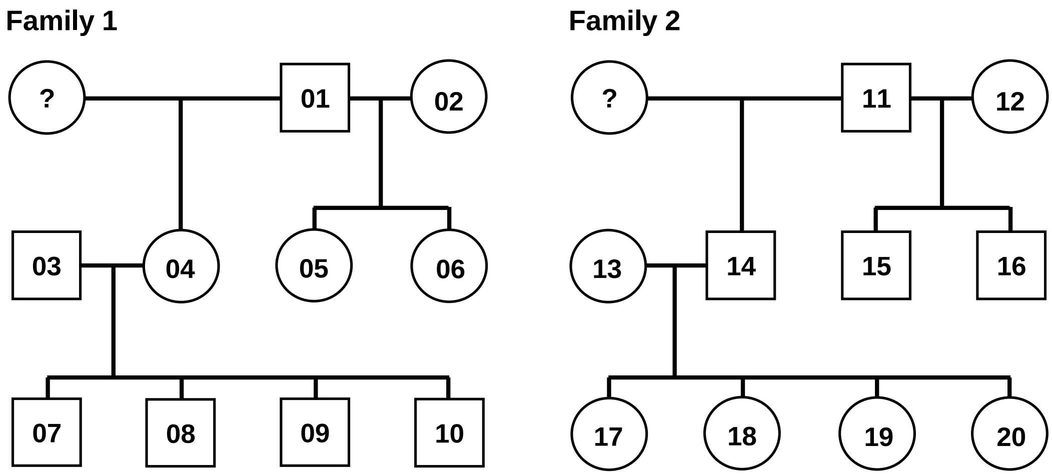

(B)

Family	Generation	Individual name	ID number	gender
Family 1	1st	S12LMC	01	Male
		131279	02	Female
	2nd	383782	03	Male
		F42MFC	04	Female
		U23PFC	05	Female
		U33PFC	06	Female
	3rd	K1YPMC	07	Male
		K2YPMC	08	Male
		K3YPMC	09	Male
		K4yPMC	10	Male
Family 2	1st	176365	11	Male
		S21OFC	12	Female
	2nd	Q57MFC	13	Female
		384323	14	Male
		T11QMC	15	Male
		T21QMC	16	Male
	3rd	Z23PFC	17	Female
		Z33PFC	18	Female
		Z43PFC	19	Female
		Z53PFC	20	Female

Supplementary figure 2. Genetic and biological information of the 20 cats. (A) shows pedigree charts of 20 cats from two families used for this study. Circles and squares indicate females and males, respectively, and numbers in the circles and squares indicate the cat's ID number. Question marks indicate individuals with unknown FLA genotypes. (B) shows individual and cat ID number and gender.
