# Supplementary material for: Identification of Novel Alleles and Structural Haplotypes of Major Histocompatibility Complex Class I and DRB Genes in Domestic Cat (Felis catus) by a Newly Developed NGS-Based Genotyping Method
Source: Front Genet. 2020 Jul 15;11:750. doi: 10.3389/fgene.2020.00750 (PMC7375346; doi:10.3389/fgene.2020.00750)
Supplement: Supplementary file 1 [file Data_Sheet_1.zip › Supplementary Figure 3.PDF]

Supplementary figure 3

(A)

```
                        FLA-I_CDS_F                                          FLA-I_Umultiple_F
         5'UTR (Exon 1)  --------->                     SP region (Exon 1)    --------->
FLA-E    1 AGTTTCCGCACCCGCCTCCTGAGACTCACATTTCTCCTCAGACCCCGAGGATGCGGTTCGTGATGTCCCCAACTGTGCTCCTGCTGCTGTTGGGGGCCCT  100
FLA-K    1 .....................................C......................C.........G.............................  100
FLA-H    1 ............G...............................................C.........G.............................  100

                                 α1 domain (Exon 2)
FLA-E  101 GGCCGCGCCCCAGACCTGGGCG|GGCTCCCACTCCCTGAGGTATTTCTACACCGCGGTGTCCCGGCCCGGCCTCGGGGAGCCCCGCTTCATCTCCGTGGGC  200
FLA-K  101 ............A.........|.................................A...................................G........  200
FLA-H  101 ......................|......................................................A..............G........  200

FLA-E  201 TACGTGGACGACACGCAGTTCGTGCGGTTCGACAGCGACGCCCCGAATCCCAGGGAAGAGCCGCGGGCGCCGTGGATGGAGCAGGAGGGGCCGGAGTATT  300
FLA-K  201 ..................................................G...ATG..................G.........T..............  300
FLA-H  201 ..................................................G...ATG...........................................  300

                                                                                                  α2 domain
FLA-E  301 GGGACCGGAACACGCGGATTTACTTGGACACCGCACAGATTTCCCGAGTGAACCTGAACACGTTGCTCCGCTACTACAACCAGAGCGAGTCC|GGGTCGCA  400
FLA-K  301 ......A.G.G.......ACG.GAA.A...A...........T.......G...........A.............................|........  400
FLA-H  301 ..................ACGCGAA.....A.............................................................|.....A..  400

           (Exon 3)
FLA-E  401 CAACATCCAGAGAATGTATGGCTGTGACGTGGACCCAGACCGGCGCTTCCTCCGCGGGTACAGTCAGGACTCCTATGACGGCAAGGATTACATCGCCCTG  500
FLA-K  401 ..........T.G...................G........................A..........................................  500
FLA-H  401 ..G.................................................................................................  500
           **.*******.*.*******************.************************.******************************************

FLA-E  501 AACGAGGACCTGCGCTCCTGGACCGCGGCGGACACCGCGGCGCAGATCACACGCCGCAAGTGGGAGGAGGCCGGTGTGGCGGAGCAGGAGAGGAACTACC  600
FLA-K  501 ....................................................................................................  600
FLA-H  501 ...A..............................................C.....................T.......C..AG.CATC..........  600
           ***.**********************************************.*********************.*******.**..*....**********

                                                                           α3 domain (Exon 4)
FLA-E  601 TGGAGGGCACGTGCGTGGAGTGGCTCGCCAAATACCTGGACATGGGGAAGGAGACGCTGCTGCGCGCA|GAATCTCCCAACACACGCGTGACCCGCCACCC  700
FLA-K  601 ....................................................................|................................  700
FLA-H  601 .....................C...A.G........................................|.........C.A..G.AG.............G  700

FLA-E  701 CATCTCTGACCGTGAGGTGACCCTGAGGTGCTGGGCCCTGGGCTTCTACCCTGCGGAGATCACCCTGACCTGGCAGCGTGATGGGCAGGACCACACCCAG  800
FLA-K  701 ....................................T...............................................................  800
FLA-H  701 G..........A......A.............................................................................T...  800

                                                                                      FLA-I_Umultiple_R
                                                                                       <---------
FLA-E  801 GACACAGAGCTTGTGGAGACCAGGCCTGCGGGAGATGGACCTTCCAGAAGTGGGCGGCTGTGGTGGTGCCTTCTGGAGAGGAGCAGAGATACACGTGCC  900
FLA-K  801 ...G....................................................A............T.............................  900
FLA-H  801 ...G...............................................................................................  900

           _
                                                          Transmembrane domain (Exon 5)
FLA-E  901 ATGTGCAGCACAAGGGGCTGCCCGAGCCCATCAACTTGAGATGG|GAGCCATCGTCTCTGCCCTTCATCACCATTCTGGGCATCATTGCTGGTGTGGCTGT 1000
FLA-K  901 ...........G.........G...........C..........|........C....A.....C....C....C.......................... 1000
FLA-H  901 ...........G..........T..........C..........|..A...ATCC...A.....C....C....C.C...G..................A. 1000

                                                  Cytoplasmic domain (Exons 6-8)
FLA-E 1001 CCTTGTGGTCACTGTGGTGGTTGGAGCTGTGATCTGG|AGGAAGAAGTGCTCAGGAGGAAAAGGACCAATCTATTCTCACGCTGCACGCGACGACAGTACC 1100
FLA-K 1001 ...........T.........................|..........T............G.......CG.................T..T........T 1100
FLA-H 1001 ...........TG......A.................|..........T....................G...C....T............T......... 1100

                                                                     FLA-I_CDS_R
                                                  3'UTR (Exon 8)     <---------
FLA-E 1101 CAGGGCTCTGATTCGTCTCTAATGGCTCCTAAAGTTTGAGACCCGCTGCCTGTGGAGAACTGAGCGATGCAGGATCTGTTCACACTCCCACTTGGTGACA 1200
FLA-K 1101 ..................G................G........A....T.....T.G............................................ 1200
FLA-H 1101 ....................C..............G...................T.C.......................TG................ 1200
```

(B)

```
                                                                                        FLA-I_F2
              α1 domain (Exon 2)
FLA-E      1 GGCTCCCACTCCCTGAGGTATTTCTACACCGCGGTGTCCCGGCCCGGCCTCGGGGAGCCCCGCTTCATCTCCGTGGGCTACGTGGACGACACGCAGTTCG   100
FLA-H      1 ......................................................A..............G..............................   100
FLA-K      1 .................................A...................................G..............................   100
FLA-A      1 ........T...T...T.......GG...................A................G.A.T.GGAA..C.................A.......   100
FLA-J      1 ........................C........A...............G................................................T.   100
FLA-L      1 .................................................G..................................................   100
FLA-O      1 ............T...........C........A......................A............G..............................   100

FLA-E    101 TGCGGTTCGACAGCGACGCCCCGAATCCCAGGGAAGAGCCGCGGGCGCCGTGGATGGAGCAGGAGGGGCCGGAGTATTGGGACCGGAACACGCGGATTTA   200
FLA-H    101 ............................G...ATG.............................................................ACGC   200
FLA-K    101 ............................G...ATG..................G.........T....................A.G.G.......ACG.   200
FLA-A    101 C...................T...G...G...ATG.....A..C.T..AT...T..A.........C.....G.........G.A.G.G......GGCGC   200
FLA-J    101 ............C........T......G...TCT.............G..............T....T.C.............A.G.G.......ACGC   200
FLA-L    101 ...................................................................................GA.C.G........GGT   200
FLA-O    101 ............C.........A.....G...ATG............................T......A...............G.G........CGC   200

                                                                                α2 domain (Exon 3)
FLA-E    201 CTTGGACACCGCACAGATTTCCCGAGTGAACCTGAACACGTTGCTCCGCTACTACAACCAGAGCGAGTCC|GGGTCGCACAACATCCAGAGAATGTATGGC   300
FLA-H    201 GAA.....A.............................................................|.....A....G...................   300
FLA-K    201 GAA.A...A...........T.......G...........A.............................|..................T.G.........   300
FLA-A    201 .AA..............C..T....C...G..........C...G.G...................AG..|.....T....C.......T.GC...C.TC.   300
FLA-J    201 GAA.....A........AA.TT.......G....C.G.ACA...G.G...................T...|..A..A............T.G.........   300
FLA-L    201 .AA....CA........C..T....C...G........ACC...G.G..........T.......T....|.A........G.C.................   300
FLA-O    201 .AA..TGCA....G.....G.....CC.......C.G..CGCC...........................|.....A....G.............C.....   300

                                                                  FLA-I_exp_F
FLA-E    301 TGTGACGTGGACCCAGACCGGCGCTTCCTCCGCGGGTACAGTCAGGACTCCTATGACGGCAAGGATTACATCGCCCTGAACGAGGACCTGCGCTCCTGGA   400
FLA-H    301 .................................................................................A..................   400
FLA-K    301 ..........G........................A................................................................   400
FLA-A    301 ..C..A....GG..C..TT.....C.......A..A...T.G...TT.G...........G..................................T....   400
FLA-J    301 ......A.C.GA..GA..A.C...C.....................TG..............A...........................G.........   400
FLA-L    301 ......A.C.GG..T...G.C...C.......................G....C......G..........................T............   400
FLA-O    301 ......A.C.GG..T...G.A...C....................TTGG....C......GC......................................   400

                  FLA-I_R2m
FLA-E    401 CCGCGGCGGACACCGCGGCGCAGATCACACGCCGCAAGTGGGAGGAGGCCGGTGTGGCGGAGCAGGAGAGGAACTACCTGGAGGGCACGTGCGTGGAGTG   500
FLA-H    401 ............................C.....................T.......C..AG.CATC...............................C   500
FLA-K    401 ....................................................................................................   500
FLA-A    401 .............G..............C................CT.....A.CA..C.....TC.A......G.....C...T...A.....A.....   500
FLA-J    401 ............................C....A..C........T....T...CT..A.....CT.C..................GT...........C   500
FLA-L    401 .............................................T........A........GCTG.....................A.........G.   500
FLA-O    401 ................T............................T........A.....C..GCT.T.C...G..........A.......T...C.G.   500

                                                        α3 domain (Exon 4)
FLA-E    501 GCTCGCCAAATACCTGGACATGGGGAAGGAGACGCTGCTGCGCGCA|GAATCTCCCAACACACGCGTGACCCGCCACCCCATCTCTGACCGTGAGGTGACC   600
FLA-H    501 ....A.G.......................................|.........C.A..G.AG.............GG..........A......A...   600
FLA-K    501 ..............................................|......................................................   600
FLA-A    501 ....CGG..G...T....G.G......CA..............A..|..CC.C..G..G....A.A.C....A.T......CT.......A...T..C...   600
FLA-J    501 ....A.............G.............G.............|.........C.A.....G....................................   600
FLA-L    501 ....C.............G...............T...........|........TG......AT.........................A..........   600
FLA-O    501 ....CG..GG........G.......................T...|..G........A....A..............GG..........A..........   600

                                          FLA-I_exp_R
FLA-E    601 CTGAGGTGCTGGGCCCTGGGCTTCTACCCTGCGGAGATCACCCTGACCTGGCAGCGTGATGGGCAGGACCACACCCAGGACACAGAGCTTGTGGAGACCA   700
FLA-H    601 ..........................................................................T......G..................   700
FLA-K    601 ..............T..................................................................G..................   700
FLA-A    601 ....A...............T.....T.................A..................G......TG.T......A..G...T.......C..T.   700
FLA-J    601 ....................................................................................................   700
FLA-L    601 ....................T...........A................................................G.............C....   700
FLA-O    601 ...............T......................................A...............................A...........G.   700
```

(C)

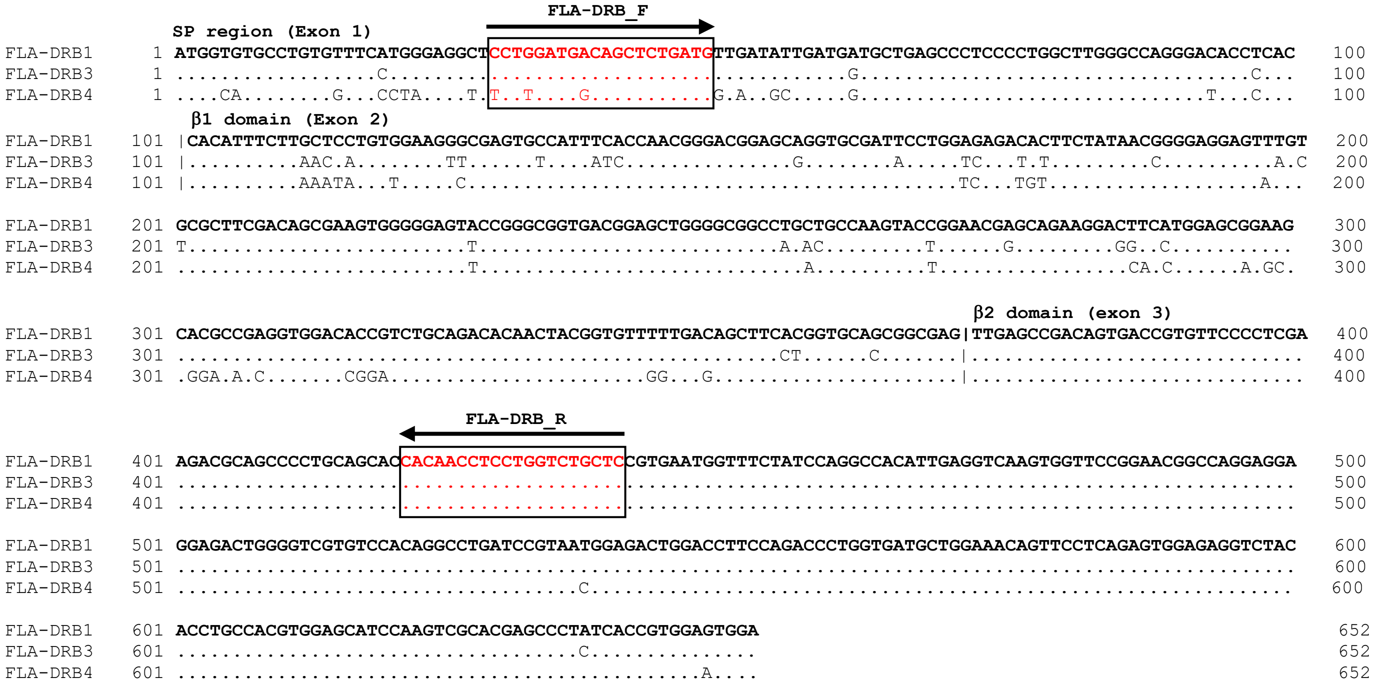

Supplementary figure 3. Nucleotide alignment for FLA-I and FLA-DRB cDNA sequences and primer locations. (A) shows the nucleotide alignment of FLA-E/H/K genes and primer locations for sub-cloning of FLA-I genes (**Table 2B**) and for confirmation of the FLA-I genotyping results (**Table 2E**), (B) shows the nucleotide alignment of seven FLA-I genes and primer locations for FLA-I expression analysis (**Table 2A**) and for genotyping of FLA-I genes (**Table 2C**), and (C) shows the nucleotide alignment of seven FLA-DRB genes and primer locations for genotyping of FLA-DRB genes (**Table 2D**). The nucleotide alignments were constructed by using the 2.98 Mb genomic sequence (EU153401). 5'UTR, SP and 3'UTR indicate 5' untranslated region, signal peptide and 3' untranslated region, respectively. Locations of primers are indicated by boxes and red letters. Arrows shows direction of primers.
