# Supplementary material for: Identification of Novel Alleles and Structural Haplotypes of Major Histocompatibility Complex Class I and DRB Genes in Domestic Cat (Felis catus) by a Newly Developed NGS-Based Genotyping Method
Source: Front Genet. 2020 Jul 15;11:750. doi: 10.3389/fgene.2020.00750 (PMC7375346; doi:10.3389/fgene.2020.00750)
Supplement: Supplementary file 1 [file Data_Sheet_1.zip › Supplementary Figure 4.PDF]

Supplementary figure 4

(A)

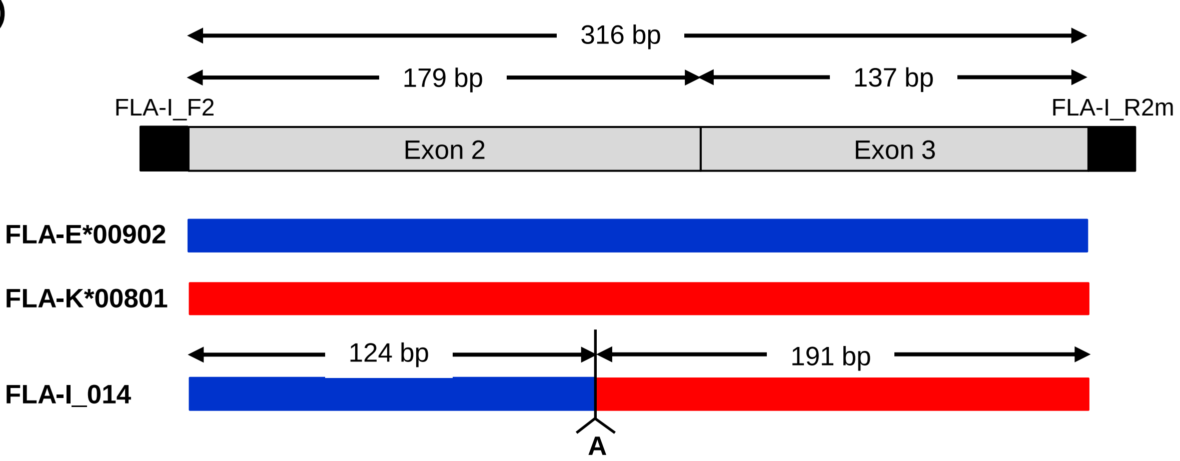

(B)

```
  1 CGCAGTTCGT GCGGTTCGAC AGCGACGCCC CGAATCCGAG GATGGAGCCG CGGGCGCCGT  60
 61 GGGTGGAGCA GGAGGGGCCG GAGTATTGGG ACCGGGAGAC GCGGAACATG AAGAACACCG 120
121 CACAAATTTT CCGAGTGAGC CTGGAGAACA CGCGCGGATA CTATAACCAG AGCGGTTCCG 180

181 GATCGCACAA CTTCCAGACA ATGTATGGCT GTGACATCGG GCCTTATGGA CGCCTCCTCC 240
241 GCGGGTACAG TCAGATGGCC TACGACGGCG CGGATTACAT CGCCCTGAAC GAGGACCTGC 300
301 GCTCCTGGAC CGCGGC 316
```

(C)

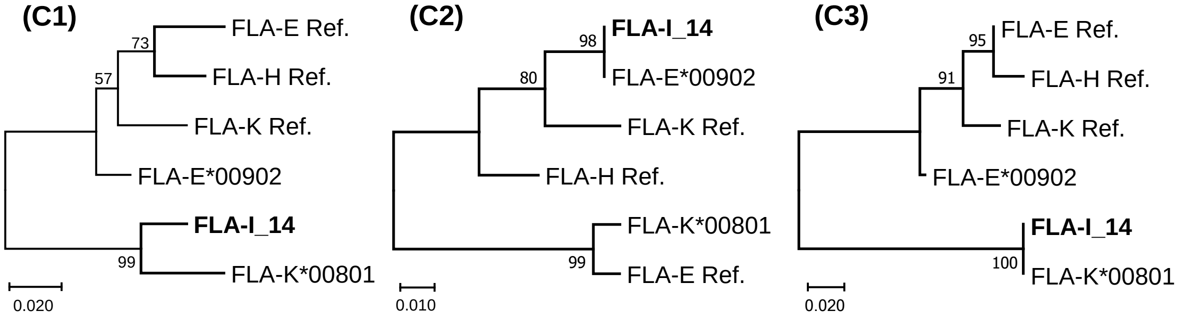

(D)

Identical to FLA-E*00902 (KC763019)		Identical to FLA-E*00902 (KC763019)		Identical to FLAI-K*00801 (KC763045)	
FLA allelic sequence	Accession	FLA allelic sequence	Accession	FLA allelic sequence	Accession
FLA-E*009:03:01:02	MN069863.1	FLA-E*009:02:01:01	MN069845	FLAB9	U07672
FLA-E*010:01:01:01	MN069861.1	FLAI-E*01101	KC763021	FLA-K*008:02:01:01Q	MK753258
FLA-E*011:01:01:01	MN069853.1	FLAI-E*01001	KC763020		
FLA-E*009:03:01:03	MN069851.1	FLAA23	U07669		
FLA-E*010:01:01:01	MN069849.1	FLAA1	U07667		
FLA-E*009:03:01:01	MN069846.1				

Supplementary figure 4. Gene structure of the recombinant FLA-I_014 sequence. (A) Schematic diagram showing the PCR region used for genotyping and the location of the recombination hotspot. Blue and red bars indicate nucleotides that support the FLA-E*00902 (KC763019) and K*00801 (KC763045) sequences, respectively. (B) Nucleotide sequence of FLA-I_014. Arrow indicates estimated recombination hotspot. (C) Phylogenetic analyses using FLA-E/H/K and FLA-I_014 sequences. The FLA-E Ref., FLA-H Ref. and FLA-K Ref were obtained from the cat genome reference sequence (EU153401). Numbers showing on the branches are bootstrap values. (C1) The 316 bp alignment for the PCR region was used. (C2) The 124 bp alignment for the exon 2 was used. (C3) The 191 bp alignment for exons 2 and 3 was used. (D) List of FLA-I alleles identical to FLA-E*00902 and K*00801.
