# Supplementary material for: Identification of Novel Alleles and Structural Haplotypes of Major Histocompatibility Complex Class I and DRB Genes in Domestic Cat (Felis catus) by a Newly Developed NGS-Based Genotyping Method
Source: Front Genet. 2020 Jul 15;11:750. doi: 10.3389/fgene.2020.00750 (PMC7375346; doi:10.3389/fgene.2020.00750)
Supplement: Supplementary file 1 [file Data_Sheet_1.zip › Supplementary Table 1.PDF]

Supplementary table 1. GeneScan analysis of conservation of coding exons in the FLA-class I loci

Locus	Conserved exon	Length of coding exon (bp)								Total length of CDS	
		exon 1	exon 2	exon 3	exon 4	exon 5	exon 6	exon 7	exon 8	nt	aa
FLA-A	exon 1-8	73	270	276	276	107*				1002	334
FLA-C	exon 2-5		269	285	21					-	-
FLA-E	exon 1-8	73	270	276	276	108	33	48	5*	1089	363
FLA-F	exon 1-8	73	270	275	21*					-	-
FLA-H	exon 1-8	73	270	276	276	108	33	48	5*	1089	363
FLA-J	exon 1-8	70	270	276	276	108	33	48	5*	1086	362
FLA-K	exon 1-8	73	270	276	276	108	33	48	5*	1089	363
FLA-L	exon 1-8	73	270	276	276	108	33	17*		1053	351
FLA-M	exon 1-8	73	270	273	276	108	33	48	5*	1086	362
FLA-O	exon 1-8	73	270	276	276	108	33	48	5*	1089	363
FLA-Q	exon 1-8	61	269*							-	-
FLA-S	exon 1-5	73	176*							-	-

Asterisk indicates the presence of the exonic terminator codon. Yellow background indicates the classical FLA-I loci that were confirmed by analysis of gene expression.
