# Supplementary material for: Identification of Novel Alleles and Structural Haplotypes of Major Histocompatibility Complex Class I and DRB Genes in Domestic Cat (Felis catus) by a Newly Developed NGS-Based Genotyping Method
Source: Front Genet. 2020 Jul 15;11:750. doi: 10.3389/fgene.2020.00750 (PMC7375346; doi:10.3389/fgene.2020.00750)
Supplement: Supplementary file 1 [file Data_Sheet_1.zip › Supplementary Table 2A.PDF]

Supplementary table 2A. Read information for genotyping of FLA-class I genes in the two cat families

(A) FLA-claass I

FLA locus lineage in Figure 2A	FLA-I sequence name*	Accession number	Family 1										Family 2									
			01	02	03	04	05	06	07	08	09	10	11	12	13	14	15	16	17	18	19	20
FLA-E/H/K	FLA-E*00501	KC763048	12179	0	0	31332	28084	26666	0	0	0	28269	0	27281	0	0	32844	0	0	0	0	0
FLA-E/H/K	FLA-E*00902	KC763019	0	0	0	18333	0	0	15585	15263	15923	0	0	0	14386	0	0	0	15681	15539	0	16352
FLA-E/H/K	FLA-E*01401	KC763050	0	0	0	0	0	0	0	0	0	0	0	28739	21472	0	0	23411	0	0	23881	0
FLA-E/H/K	FLA-E*01801	EU915360	0	7504	0	0	11645	0	0	0	0	0	0	0	0	0	0	0	0	0	0	0
FLA-E/H/K	FLA-H*003011	KC763028	20171	0	0	0	0	0	0	0	0	0	0	0	0	0	0	0	0	0	0	0
FLA-E/H/K	FLA-H*008011	KC763034	0	0	0	0	0	0	0	0	0	0	0	10619	10161	0	0	10520	0	0	8902	0
FLA-E/H/K	FLA-K*00101	EU153401	0	0	7503	0	0	0	8222	7780	0	6874	0	0	0	0	0	0	0	0	0	0
FLA-E/H/K	FLA-K*00303	KC763039	0	0	0	0	0	0	0	0	0	0	0	13133	10876	0	0	11741	0	0	11518	0
FLA-E/H/K	FLA-K*00401	KC763049	4628	0	0	0	0	0	0	0	0	0	0	0	0	0	0	0	0	0	0	0
FLA-E/H/K	FLA-K*00701	KC763047	12897	0	0	29436	8973	8850	19177	19956	23379	8280	0	8127	24780	0	9686	0	23677	24124	0	23005
FLA-E/H/K	**FLA-I_001**	**LC534228**	0	29099	20896	0	0	29111	0	0	25226	0	50725	0	0	54450	28380	23536	23101	21507	23886	22439
FLA-E/H/K	**FLA-I_002**	**LC534229**	0	4647	6977	0	0	8131	0	0	7592	0	15354	0	0	15737	7485	8708	8031	6918	7031	6991
FLA-E/H/K	**FLA-I_003**	**LC534230**	0	0	24315	0	0	0	16509	18921	0	20260	0	0	0	0	0	0	0	0	0	0
FLA-E/H/K	**FLA-I_004**	**LC534231**	0	31513	0	0	24697	0	0	0	0	0	0	0	0	0	0	0	0	0	0	0
FLA-E/H/K	**FLA-I_005**	**LC534232**	0	12292	0	0	12557	0	0	0	0	0	0	0	0	0	0	0	0	0	0	0
FLA-E/H/K	**FLA-I_006**	**LC534233**	39954	0	0	0	0	0	0	0	0	0	0	0	0	0	0	0	0	0	0	0
FLA-E/H/K	FLA-H*016:01	MK644232	0	0	14883	0	0	0	12992	11959	0	11901	0	0	0	0	0	0	0	0	0	0
FLA-E/H/K	**FLA-I_007**	**LC534234**	0	0	8604	0	0	0	8240	8757	0	10546	0	0	0	0	0	0	0	0	0	0
FLA-E/H/K_Rec	**FLA-I_014**	**LC534241**	0	0	0	14090	0	0	12972	12564	9328	0	0	0	11328	0	0	0	10335	11959	0	11987
FLA-A	FLA-A	EU153401	369	16	511	0	483	482	371	287	129	471	205	0	0	79	170	226	112	100	213	182
FLA-J	FLA-E*01601	EU915358	7483	474	0	6466	7975	6803	835	886	676	8283	0	5637	788	0	5825	0	809	832	0	972
FLA-J	**FLA-I_008**	**LC534235**	0	5161	5798	0	0	10092	0	0	8185	0	17388	0	0	14284	7628	8652	7465	8830	8925	8002
FLA-J	FLA-J*01:18	MK737915	0	3609	4260	0	0	6061	0	0	6272	0	11558	0	0	10565	4711	5418	7504	7409	7125	7299
FLA-J	**FLA-I_009**	**LC534236**	0	0	3202	0	0	0	3613	2495	0	4101	0	0	0	0	0	0	0	0	0	0
FLA-J	FLA-J*01:16	MK737902	0	0	0	0	0	0	0	0	0	0	0	4942	4567	0	0	3756	0	0	5468	0
FLA-J	FLA-J*01:14	MK737908	229	1293	0	0	3256	0	0	0	0	0	0	0	0	0	0	0	0	0	0	0
FLA-L	FLA-L	EU153401	41	682	236	0	0	139	479	319	472	196	503	128	385	304	180	517	326	273	258	245
FLA-L	**FLA-I_013**	**LC534240**	0	364	0	0	154	0	0	0	0	0	0	0	0	0	0	0	0	0	0	0
FLA-O	FLA-O	EU153401	1527	1963	1448	209	1644	1481	1004	813	1327	332	1337	0	481	1281	693	593	1150	1054	868	913
FLA-O	**FLA-I_010**	**LC534237**	0	1383	1366	0	0	1584	0	0	1491	0	2930	0	0	3301	1437	1758	1809	1454	1196	1613
FLA-O	**FLA-I_011**	**LC534238**	0	0	0	0	0	0	0	0	0	0	0	782	776	0	0	1163	0	0	727	0
FLA-O	**FLA-I_012**	**LC534239**	523	0	0	134	532	599	0	0	0	486	0	611	0	0	960	0	0	0	0	0
Total sequence number			11	14	13	7	11	12	12	12	12	12	8	10	11	8	12	13	12	12	13	12

The read numbers are normalized per 100,000 reads per cat. Novel FLA-class I sequences and their accession numbers are indicated by bold letters.
