# Supplementary material for: Identification of Novel Alleles and Structural Haplotypes of Major Histocompatibility Complex Class I and DRB Genes in Domestic Cat (Felis catus) by a Newly Developed NGS-Based Genotyping Method
Source: Front Genet. 2020 Jul 15;11:750. doi: 10.3389/fgene.2020.00750 (PMC7375346; doi:10.3389/fgene.2020.00750)
Supplement: Supplementary file 1 [file Data_Sheet_1.zip › Supplementary Table 2B.PDF]

Supplementary table 2B. Read information for genotyping of FLA-DRB genes in the two cat families

(B) FLA-DRB

FLA locus lineage in Figure 2B	FLA-DRB sequence name*	Accession number	Family 1										Family 2									
			01	02	03	04	05	06	07	08	09	10	11	12	13	14	15	16	17	18	19	20
FLA-DRB1	FLA-DRB*0401*	U51527	0	0	0	0	0	0	0	0	0	0	0	40942	40767	0	0	40982	0	0	62614	0
FLA-DRB1	FLA-DRB*n05	EU916196	19746	23726	20467	18568	24202	43502	0	0	18136	22276	0	21414	0	22137	24011	0	14994	0	0	0
FLA-DRB1	**FLA-DRB_004**	**LC534245**	0	13859	0	0	6994	0	0	0	0	0	0	0	0	0	0	0	0	0	0	0
FLA-DRB3	FLA-DRB*0203*	U51498	39908	0	0	0	0	0	0	0	0	0	0	0	0	0	0	0	0	0	0	0
FLA-DRB3	FLA-DRB*n06	EU916197	16105	0	0	14127	22868	17103	0	0	0	17357	0	13763	0	0	18845	0	0	0	0	0
FLA-DRB3	**FLA-DRB_001**	**LC534242**	11542	24149	0	0	19322	0	0	0	0	0	0	0	0	0	0	0	0	0	0	0
FLA-DRB3	**FLA-DRB_002**	**LC534243**	0	0	0	13138	0	0	12916	12737	13842	0	0	0	8013	0	0	0	7712	11005	0	7439
FLA-DRB3	**FLA-DRB_003**	**LC534244**	0	0	0	0	0	0	0	0	0	0	0	12579	6083	0	0	14556	0	0	10668	0
FLA-DRB3	**FLA-DRB_006**	**LC534247**	0	8051	32123	0	0	15650	13464	13327	12958	15438	0	0	0	18892	0	0	9464	0	0	0
FLA-DRB4	FLA-DRB*0103*	U51483	0	0	0	7309	0	0	8702	7273	9911	0	0	0	10949	0	0	0	10961	12658	0	9073
FLA-DRB4	FLA-DRB*0107*	U51487	12700	30215	11432	7659	26615	23745	0	0	7672	13708	0	9194	0	11277	12321	0	12783	0	0	0
FLA-DRB4	FLA-DRB*0301*	U51514	0	0	2006	0	0	0	1394	2784	0	3569	0	2108	2922	0	0	2322	0	0	1772	0
FLA-DRB4	FLA-DRB1-rr6*	AJ428211	0	0	0	0	0	0	0	0	0	0	81776	0	0	36757	36075	35246	0	18537	18371	22485
FLA-DRB4	FLA-DRB1-rr7*	AJ428212	0	0	0	0	0	0	0	0	0	0	18224	0	0	10937	8748	6893	0	7825	6575	9040
FLA-DRB4	**FLA-DRB_007**	**LC534248**	0	0	33972	0	0	0	23735	24796	0	27651	0	0	0	0	0	0	0	0	0	0
FLA-DRB5	**FLA-DRB_005**	**LC534246**	0	0	0	39199	0	0	39788	39084	37481	0	0	0	31266	0	0	0	44086	49975	0	51963
Total sequence number			5	5	5	6	5	4	6	6	6	6	2	6	6	5	5	5	6	5	5	5

The read numbers are normalized per 100,000 reads per cat. Novel FLA-DRB sequences and their accession numbers are indicated by bold letters. Asterisks show the allele sequences with 237-238 bp of exon 2 in FLA-DRB gene.
