# Supplementary material for: Identification of Novel Alleles and Structural Haplotypes of Major Histocompatibility Complex Class I and DRB Genes in Domestic Cat (Felis catus) by a Newly Developed NGS-Based Genotyping Method
Source: Front Genet. 2020 Jul 15;11:750. doi: 10.3389/fgene.2020.00750 (PMC7375346; doi:10.3389/fgene.2020.00750)
Supplement: Supplementary file 1 [file Data_Sheet_1.zip › Supplementary Table 3.PDF]

Supplementary table 3. Estimated FLA-I and FLA-DRB haplotypes

Simplified name	01		02		03		04		05	
FLA-class I Hp.	Hp-1.0	Hp-2.0	Hp-3.0	Hp-4.0	Hp-3.0	Hp-7.0	Hp-1.0	Hp5.0	Hp-1.0	Hp-4.0
FLA-E/H/K	E*00501<br>K*00701	H*003011<br>K*00401<br>FLAI_006	FLAI_001<br>FLAI_002	E*01801<br>FLAI_004<br>FLAI_005	FLAI_001<br>FLAI_002	K*00101<br>FLAI_003<br>H*016:01<br>FLAI_007	E*00501<br>K*00701	E*00902<br>K*00701	E*00501<br>K*00701	E*01801<br>FLAI_004<br>FLAI_005
FLA-E/H/K_Rec								FLAI_014		
FLA-A		FLA-A	FLA-A	FLA-A	FLA-A	FLA-A				FLA-A
FLA-J	E*01601	J*01:14	FLAI_008<br>J*01:18	E*01601<br>J*01:14	FLAI_008<br>J*01:18	FLAI_009	E*01601	E*01601	E*01601	E*01601<br>J*01:14
FLA-L		FLA-L	FLA-L	FLAI_013	FLA-L	FLA-L				FLAI_013
FLA-O	FLAI_012	FLA-O	FLA-O<br>FLAI_010	FLA-O	FLA-O<br>FLAI_010	FLA-O	FLAI_012	FLA-O	FLAI_012	FLA-O
FLA-DRB Hp.	Hp-0.1	Hp-0.2	Hp-0.8	Hp-0.4	Hp-0.8	Hp-0.7	Hp-0.1	Hp-0.5	Hp-0.1	Hp-0.4
FLA-DRB1	DRB*n05		DRB*n05	DRB_004	DRB*n05		DRB*n05		DRB*n05	DRB_004
FLA-DRB3	DRB*n06	DRB*0203<br>DRB_001	DRB_006	DRB_001	DRB_006	DRB_006	DRB*n06	DRB_002	DRB*n06	DRB_001
FLA-DRB4	DRB*0107		DRB*0107		DRB*0107	DRB*0301<br>DRB_007	DRB*0107	DRB*0103	DRB*0107	
FLA-DRB5								DRB_005		

Simplified name	06		07		08		09		10	
FLA-class I Hp.	Hp-1.0	Hp-3.0	Hp5.0	Hp-7.0	Hp5.0	Hp-7.0	Hp-3.0	Hp5.0	Hp-1.0	Hp-7.0
FLA-E/H/K	E*00501<br>K*00701	FLAI_001<br>FLAI_002	E*00902<br>K*00701	K*00101<br>FLAI_003<br>H*016:01<br>FLAI_007	E*00902<br>K*00701	K*00101<br>FLAI_003<br>H*016:01<br>FLAI_007	FLAI_001<br>FLAI_002	E*00902<br>K*00701	E*00501<br>K*00701	K*00101<br>FLAI_003<br>H*016:01<br>FLAI_007
FLA-E/H/K_Rec			FLAI_014		FLAI_014			FLAI_014		
FLA-A		FLA-A		FLA-A		FLA-A	FLA-A			FLA-A
FLA-J	E*01601	FLAI_008<br>J*01:18	E*01601	FLAI_009	E*01601	FLAI_009	FLAI_008<br>J*01:18	E*01601	E*01601	FLAI_009
FLA-L		FLA-L		FLA-L		FLA-L	FLA-L			FLA-L
FLA-O	FLAI_012	FLA-O<br>FLAI_010	FLA-O	FLA-O	FLA-O	FLA-O	FLA-O<br>FLAI_010	FLA-O	FLAI_012	FLA-O
FLA-DRB Hp.	Hp-0.1	Hp-0.8	Hp-0.5	Hp-0.7	Hp-0.5	Hp-0.7	Hp-0.8	Hp-0.5	Hp-0.1	Hp-0.7
FLA-DRB1	DRB*n05	DRB*n05					DRB*n05		DRB*n05	
FLA-DRB3	DRB*n06	DRB_006	DRB_002	DRB_006	DRB_002	DRB_006	DRB_006	DRB_002	DRB*n06	DRB_006
FLA-DRB4	DRB*0107	DRB*0107	DRB*0103	DRB*0301<br>DRB_007	DRB*0103	DRB*0301<br>DRB_007	DRB*0107	DRB*0103	DRB*0107	DRB*0301<br>DRB_007
FLA-DRB5			DRB_005		DRB_005			DRB_005		

Simplified name	11		12		13		14		15	
FLA-class I Hp.	Hp-3.0	Hp-3.0	Hp-1.0	Hp-6.0	Hp5.0	Hp-6.0	Hp-3.0	Hp-3.0	Hp-1.0	Hp-3.0
FLA-E/H/K	FLAI_001<br>FLAI_002	FLAI_001<br>FLAI_002	E*00501<br>K*00701	E*01401<br>H*008011<br>K*00303	E*00902<br>K*00701	E*01401<br>H*008011<br>K*00303	FLAI_001<br>FLAI_002	FLAI_001<br>FLAI_002	E*00501<br>K*00701	FLAI_001<br>FLAI_002
FLA-E/H/K_Rec					FLAI_014					
FLA-A	FLA-A	FLA-A					FLA-A	FLA-A		FLA-A
FLA-J	FLAI_008<br>J*01:18	FLAI_008<br>J*01:18	E*01601	J*01:16	E*01601	J*01:16	FLAI_008<br>J*01:18	FLAI_008<br>J*01:18	E*01601	FLAI_008<br>J*01:18
FLA-L	FLA-L	FLA-L		FLA-L		FLA-L	FLA-L	FLA-L		FLA-L
FLA-O	FLA-O<br>FLAI_010	FLA-O<br>FLAI_010	FLAI_012	FLAI_011	FLA-O	FLAI_011	FLA-O<br>FLAI_010	FLA-O<br>FLAI_010	FLAI_012	FLA-O<br>FLAI_010
FLA-DRB Hp.	Hp-0.3	Hp-0.3	Hp-0.1	Hp-0.6	Hp-0.5	Hp-0.6	Hp-0.3	Hp-0.8	Hp-0.1	Hp-0.3
FLA-DRB1			DRB*n05	DRB*0401		DRB*0401		DRB*n05	DRB*n05	
FLA-DRB3			DRB*n06	DRB_003	DRB_002	DRB_003		DRB_006	DRB*n06	
FLA-DRB4	DRB1-rr6<br>DRB1-rr7	DRB1-rr6<br>DRB1-rr7	DRB*0107	DRB*0301	DRB*0103	DRB*0301	DRB1-rr6<br>DRB1-rr7	DRB*0107	DRB*0107	DRB1-rr6<br>DRB1-rr7
FLA-DRB5					DRB_005					

Simplified name	16		17		18		19		20	
FLA-class I Hp.	Hp-3.0	Hp-6.0	Hp-3.0	Hp5.0	Hp-3.0	Hp5.0	Hp-3.0	Hp-6.0	Hp-3.0	Hp5.0
FLA-E/H/K	FLAI_001<br>FLAI_002	E*01401<br>H*008011<br>K*00303	FLAI_001<br>FLAI_002	E*00902<br>K*00701	FLAI_001<br>FLAI_002	E*00902<br>K*00701	FLAI_001<br>FLAI_002	E*01401<br>H*008011<br>K*00303	FLAI_001<br>FLAI_002	E*00902<br>K*00701
FLA-E/H/K_Rec				FLAI_014		FLAI_014				FLAI_014
FLA-A	FLA-A		FLA-A		FLA-A		FLA-A		FLA-A	
FLA-J	FLAI_008<br>J*01:18	J*01:16	FLAI_008<br>J*01:18	E*01601	FLAI_008<br>J*01:18	E*01601	FLAI_008<br>J*01:18	J*01:16	FLAI_008<br>J*01:18	E*01601
FLA-L	FLA-L	FLA-L	FLA-L		FLA-L		FLA-L	FLA-L	FLA-L	
FLA-O	FLA-O<br>FLAI_010	FLAI_011	FLA-O<br>FLAI_010	FLA-O	FLA-O<br>FLAI_010	FLA-O	FLA-O<br>FLAI_010	FLAI_011	FLA-O<br>FLAI_010	FLA-O
FLA-DRB Hp.	Hp-0.3	Hp-0.6	Hp-0.8	Hp-0.5	Hp-0.3	Hp-0.5	Hp-0.3	Hp-0.6	Hp-0.3	Hp-0.5
FLA-DRB1		DRB*0401	DRB*n05					DRB*0401		
FLA-DRB3		DRB_003	DRB_006	DRB_002		DRB_002		DRB_003		DRB_002
FLA-DRB4	DRB1-rr6<br>DRB1-rr7	DRB*0301	DRB*0107	DRB*0103	DRB1-rr6<br>DRB1-rr7	DRB*0103	DRB1-rr6<br>DRB1-rr7	DRB*0301	DRB1-rr6<br>DRB1-rr7	DRB*0103
FLA-DRB5				DRB_005		DRB_005				DRB_005

Red letter indicates novel allele.
