# Supplementary material for: Identification of Novel Alleles and Structural Haplotypes of Major Histocompatibility Complex Class I and DRB Genes in Domestic Cat (Felis catus) by a Newly Developed NGS-Based Genotyping Method
Source: Front Genet. 2020 Jul 15;11:750. doi: 10.3389/fgene.2020.00750 (PMC7375346; doi:10.3389/fgene.2020.00750)
Supplement: Supplementary file 1 [file Data_Sheet_1.zip › Supplementary Table 4.pdf]

Supplementary table 4. Comparison of the FLA-E/H/K genotyping results between the two primer pairs designed in different locations

Simplified name	01				02				07				13			
FLA-I haplotype	Hp-1.0		Hp-2.0		Hp-3.0		Hp-4.0		Hp-5.0		Hp-7.0		Hp-5.0		Hp-6.0	
Primer pair	C	E	C	E	C	E	C	E	C	E	C	E	C	E	C	E
FLA-E*00501	13558	31728														
FLA-K*00701	14357	22362							23756	28257			30340	27838		
FLA-E*00902									19307	16750			17614	14005		
FLA-H*003011			22455	19304												
FLA-K*00401			5152	5529												
FLA-I_006			44477	21077												
FLA-I_001					34212	14833										
FLA-I_002					5464	9117										
FLA-E*01801							8822	12619								
FLA-I_004							37050	47536								
FLA-I_005							14452	15895								
FLA-H*016:01											16093	26590				
FLA-K*00101											10185	19900				
FLA-I_003											20451	7498				
FLAI_007											10207	1005				
FLA-E*01401															26290	26074
FLA-H*008011															12440	24505
FLA-K*00303															13317	7579

The read numbers are normalized per 100,000 reads per cat. "C" and "E" indicate read numbers obtained from amplicons using FLA-I primer pairs for genotyping of FLA-I genes (**Table 2C**) and for conformation of the FLA-I genotyping results (**Table 2E**), respectively. Relationships between the simplified names and FLA-I haplotypes are shown in **Figures 3** and **4**.
