# Supplementary material for: Identification of Novel Alleles and Structural Haplotypes of Major Histocompatibility Complex Class I and DRB Genes in Domestic Cat (Felis catus) by a Newly Developed NGS-Based Genotyping Method
Source: Front Genet. 2020 Jul 15;11:750. doi: 10.3389/fgene.2020.00750 (PMC7375346; doi:10.3389/fgene.2020.00750)
Supplement: Supplementary file 1 [file Data_Sheet_1.zip › Supplementary Table 5.PDF]

Supplementary table 5. Average read numbers in each gene and lineage

FLA sequence	For each gene			FLA lineage	For each lineage			
	Sequence frequency	Average reads	±SD		Sequence Num.	Sequence frequency	Average reads	±SD
**(A) FLA-I gene**								
FLA-E*00501	7	26,665	6,265	FLA-E/H/K	18	95	16,444	8,136
FLA-E*00902	8	15,883	1,066					
FLA-E*01401	4	24,376	2,676					
FLA-E*01801	2	9,575	2,071					
FLA-H*003011	1	20,171	0					
FLA-H*008011	4	10,050	685					
FLA-K*00101	4	7,595	489					
FLA-K*00303	4	11,817	823					
FLA-K*00401	1	4,628	0					
FLA-K*00701	15	16,290	6,350					
FLA-I_001	14	25,168	2,639					
FLA-I_002	14	7,400	909					
FLA-I_003	4	20,001	2,830					
FLA-I_004	2	28,105	3,408					
FLA-I_005	2	12,425	132					
FLA-I_006	1	39,954	0					
FLA-H*016:01	4	12,934	1,206					
FLAI_007	4	9,037	892					
FLA-I_014	8	11,820	1,403	FLA-E/H/K_Rec	1	8	11,820	1,403
FLA-A	21	210	152	FLA-A	1	21	210	152
FLA-E*01601	17	3,220	2,636	FLA-J	6	56	5,069	2,648
FLA-I_008	14	7,886	1,253					
FLA-J*01:18	14	5,842	1,159					
FLA-I_009	4	3,363	589					
FLA-J*01:16	4	4,683	624					
FLA-J*01:14	3	1,593	1,254					
FLA-L	23	247	144	FLA-L	2	25	248	142
FLA-I_013	2	259	105					
FLA-O	30	671	351	FLA-O	4	54	878	475
FLA-I_010	14	1,523	159					
FLA-I_011	4	862	175					
FLA-I_012	7	550	225					
**(B) FLA-DRB genes**								
FLA-DRB*0401	4	46,326	9,404	FLA-DRB1	3	19	25,228	12,376
FLA-DRB*n05	13	21,014	2,527					
FLA-DRB_004	2	10,426	3,433					
FLA-DRB*0203	1	39,908	0	FLA-DRB3	6	33	14,701	6,085
FLA-DRB*n06	7	17,167	2,861					
FLA-DRB_001	3	18,338	5,193					
FLA-DRB_002	8	10,850	2,539					
FLA-DRB_003	4	10,972	3,139					
FLA-DRB_006	10	13,937	3,082					
FLA-DRB*0103	8	9,605	1,763	FLA-DRB4	6	49	14,340	10,954
FLA-DRB*0107	13	13,794	6,528					
FLA-DRB*0301	8	2,360	654					
FLA-DRB1-rr6	8	31,156	9,080					
FLA-DRB1-rr7	8	8,530	1,312					
FLA-DRB_007	4	27,538	3,981					
FLA-DRB_005	8	41,605	6,353	FLA-DRB5	1	8	41,605	6,353
